# Human MAIT cells inhibit alloreactive T cell responses and protect against acute graft-versus-host disease

**DOI:** 10.1172/jci.insight.166310

**Published:** 2024-02-01

**Authors:** Nana Talvard-Balland, Marion Lambert, Mathieu F. Chevalier, Norbert Minet, Marion Salou, Marie Tourret, Armelle Bohineust, Idan Milo, Véronique Parietti, Thomas Yvorra, Gérard Socié, Olivier Lantz, Sophie Caillat-Zucman

**Affiliations:** 1 INSERM UMR-976 HIPI, Saint Louis Research Institute, Université Paris Cité, Paris, France.; 2 Institut Curie, Université PSL, INSERM U932, Immunity and Cancer, Paris, France.; 3 Université Paris Cité, INSERM, CNRS, UMS Saint-Louis (US53/UAR2030), Paris, France.; 4 Institut Curie, Université PSL, CNRS UMR3666, INSERM U1143, Paris, France.; 5 Hematology Transplantation, Hôpital Saint-Louis, Assistance Publique-Hôpitaux de Paris (AP-HP), Université Paris Cité, Paris, France.; 6 Clinical Immunology Laboratory, Institut Curie, Paris, France.; 7 Centre d’investigation Clinique en Biothérapie Gustave-Roussy Institut Curie (CIC-BT1428), Paris, France.; 8 Immunology Laboratory, Hôpital Saint-Louis, AP-HP, Université Paris Cité, Paris, France.

**Keywords:** Immunology, Transplantation, Stem cell transplantation, T cells

## Abstract

Adoptive transfer of immunoregulatory cells can prevent or ameliorate graft-versus-host disease (GVHD), which remains the main cause of nonrelapse mortality after allogeneic hematopoietic stem cell transplantation. Mucosal-associated invariant T (MAIT) cells were recently associated with tissue repair capacities and with lower rates of GVHD in humans. Here, we analyzed the immunosuppressive effect of MAIT cells in an in vitro model of alloreactivity and explored their adoptive transfer in a preclinical xenogeneic GVHD model. We found that MAIT cells, whether freshly purified or short-term expanded, dose-dependently inhibited proliferation and activation of alloreactive T cells. In immunodeficient mice injected with human PBMCs, MAIT cells greatly delayed GVHD onset and decreased severity when transferred early after PBMC injection but could also control ongoing GVHD when transferred at delayed time points. This effect was associated with decreased proliferation and effector function of human T cells infiltrating tissues of diseased mice and was correlated with lower circulating IFN-γ and TNF-α levels and increased IL-10 levels. MAIT cells acted partly in a contact-dependent manner, which likely required direct interaction of their T cell receptor with MHC class I–related molecule (MR1) induced on host-reactive T cells. These results support the setup of clinical trials using MAIT cells as universal therapeutic tools to control severe GVHD or mucosal inflammatory disorders.

## Abstract 2

Mucosal-associated invariant T cells have&nbsp;an intrinsic regulatory function that suppresses alloreactive T cell responses that protect against GVHD in vitro and in vivo.

## Graphical abstract

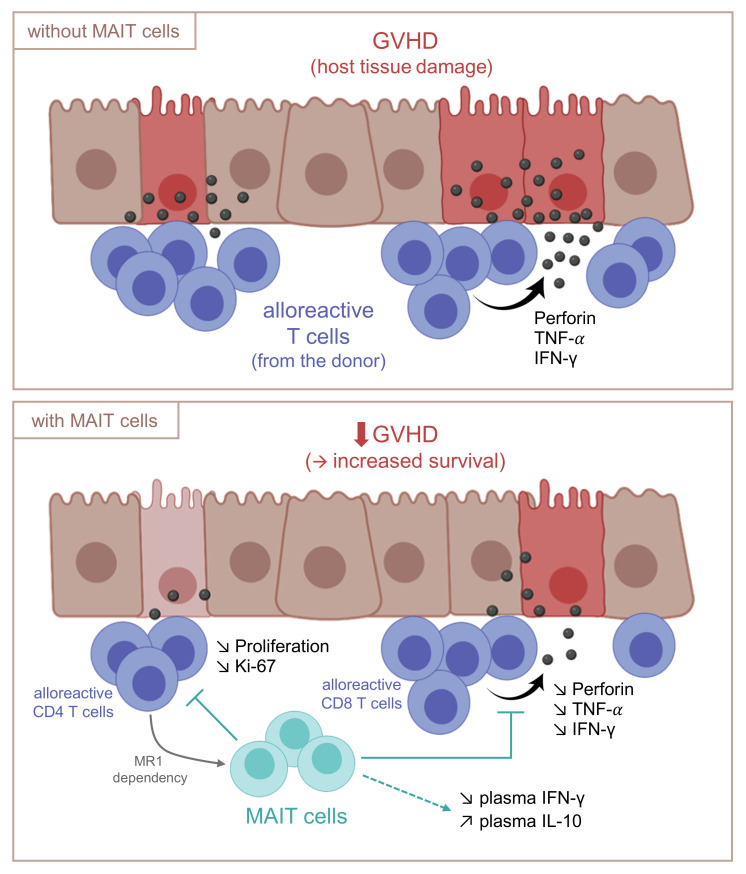

## Introduction

Allogeneic hematopoietic stem cell transplantation (allo-HCT) is a curable treatment option for many hematological malignancies, through the graft-versus-leukemia (GVL) effect mediated by donor-derived effector T cells. However, allo-HCT is frequently complicated by graft-versus-host disease (GVHD), which remains the main cause of nonrelapse mortality and morbidity. GVHD occurs when donor-derived effector T cells recognize normal nonhematopoietic cells and damage recipient tissues, such as gut, lung, liver, and skin. Activation and expansion of donor T cells lead to the secretion of proinflammatory cytokines and the recruitment of additional inflammatory effector cells to these sites, further damaging target tissues (1). Prevention and treatment of GVHD have long been limited to depletion or functional inactivation of donor T cells, but these immunosuppressive strategies are hampered by the increased risk of infections and of tumor relapse.

Adoptive transfer of immunoregulatory cells is a new approach to prevent or ameliorate GVHD while preserving transplant tolerance and tumor control. Preclinical studies have demonstrated the efficacy of various immunoregulatory populations to control immune homeostasis, reduce deleterious T cell allogeneic responses, and facilitate tissue repair (reviewed in refs. 2, 3). To date, the most appropriate regulatory lineage to be used for this purpose remains a matter of debate because of challenges in obtaining sufficient numbers of a highly purified cell population and in maintaining its survival, regulatory functions, and homing properties after transfer into recipients, without affecting the therapeutic GVL effect of allo-HCT (1, 4).

Mucosal-associated invariant T cells (MAIT cells) are innate-like T cells that express a semi-invariant T cell receptor (TCR) (Vα7.2-Ja33/20/12 in humans, combined to a limited set of Vβ chains) restricted by the monomorphic, highly conserved, MHC class I–related (MR1) molecule (5). In contrast to conventional T cells that recognize classical MHC-peptide complexes, MAIT cells recognize microbially derived riboflavin (vitamin B_2_) precursor derivatives, such as 5-(2-oxopropylideneamino)-6-d-ribitylaminouracil (5-OP-RU), presented by the MR1 molecule (6, 7). Upon recognition of activating MR1 ligands, MAIT cells display immediate potent effector responses through secretion of inflammatory cytokines (IFN-γ, TNF-α, IL-17) and perforin/granzyme-dependent cytotoxicity (8, 9). Since most bacteria and yeasts but not animal cells can synthesize riboflavin, this recognition pathway represents a discriminatory mechanism to target microbial antigens while sparing the host. In addition to riboflavin precursor products, other activating and nonactivating MR1-binding ligands have been identified, including the nonstimulatory folic acid derivative 6-formyl-pterin (Ac-6-FP), and various drugs or drug-like molecules, but their clinical relevance remains to be elucidated (10). MAIT cells can also be activated in a TCR-independent fashion in response to cytokines, such as IL-12 and IL-18 (11). An important feature of MAIT cells is their intimate relationship and localization within barrier tissues, especially the liver and mucosae (including lung and intestine) (12), presumably due to the enrichment of metabolite antigens at these sites where bacteria interface with the host.

In addition to playing a role in frontline protection of tissues against pathogens, MAIT cells recently emerged as having important regulatory and tissue repair capacities (13–16). In mouse models of allo-HCT, MAIT cells indirectly protect against acute intestinal GVHD via IL-17–mediated control of microbial translocation, thus attenuating pathogenic proinflammatory T cell responses (17). However, such mouse models are not directly translatable to human GVHD because of fundamental interspecies differences in the transplantation procedures and in the numbers and characteristics of MAIT cells (18–20). In humans, higher MAIT cell numbers early posttransplant have been associated with lower rates of GVHD (21–23). Notably, a recent study has linked a diverse intestinal microbiome early after allo-HCT with increased numbers of peripheral blood MAIT cells, which are in turn associated with less GVHD and improved overall survival (24). We recently demonstrated that human MAIT cells lack alloreactive potential and do not participate in acute GVHD (aGVHD) tissue damage in preclinical models and human HCT recipients (25, 26). Taken together, these data led us to question whether MAIT cells could be exploited as an immune adoptive therapy strategy to control GVHD. Using in vitro and in vivo preclinical models, we show here that human MAIT cells can prevent and treat GVHD via inhibition of host-reactive T cell proliferation, activation, and accumulation in target tissues and modulation of their effector functions.

## Results

### Human MAIT cells dose-dependently inhibit the in vitro proliferation and activation of alloreactive T cells.

**Figure 1:**
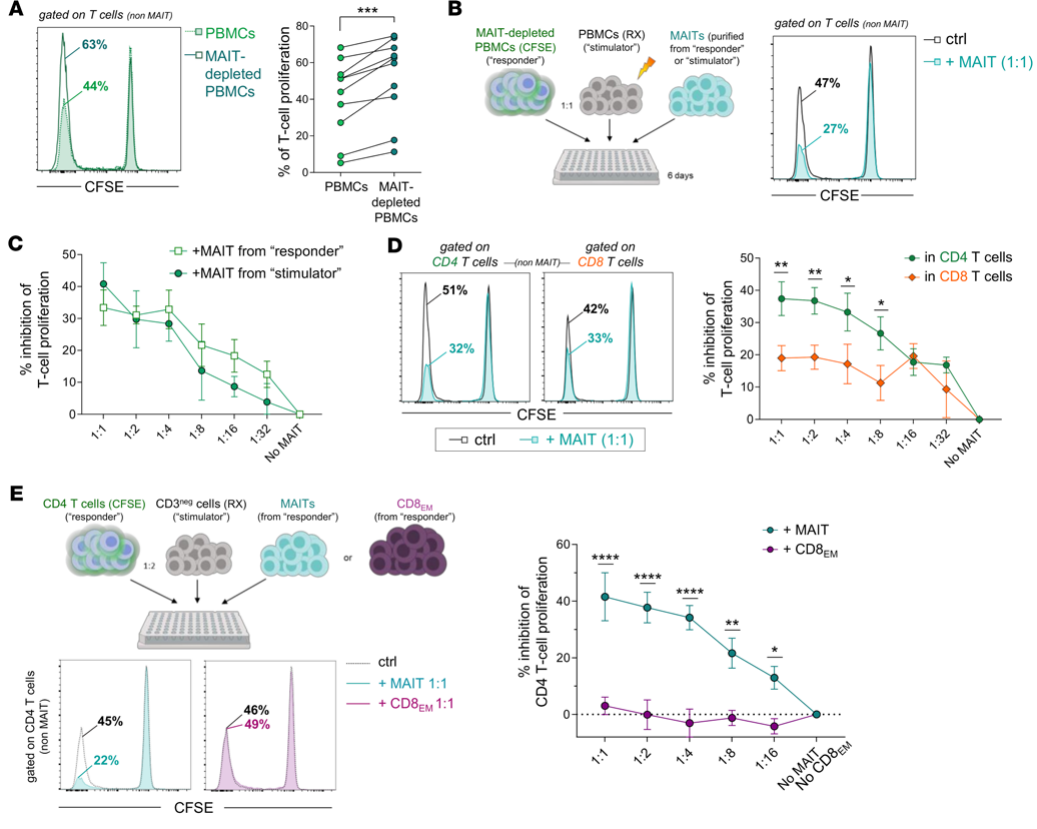
Human MAIT cells dose-dependently inhibit the in vitro proliferation of alloreactive T cells. (**A**) MLR was performed using total or MAIT-depleted CFSE-labeled PBMCs (“responders”) cocultured for 6 days with irradiated allogeneic PBMCs (“stimulators”). Proliferation was quantified as the percentage of CFSE^lo^ cells in non-MAIT (Vα7.2^–^tetramer^–^) responder T cells on day 6. Left panel shows a representative example, and the graph indicates the percentage of proliferating T cells in paired total or MAIT-depleted PBMCs (*n* = 10 different recipient/donor pairs). (**B**–**D**) FACS-sorted purified MAIT cells were added to the MLR at different MAIT/MAIT-depleted PBMC ratios. (**B**) Representative staining in the absence (ctrl) or presence of MAIT cells at 1:1 ratio. (**C**) Percentage of inhibition of responder T cell proliferation in the presence (versus absence) of MAIT cells purified from autologous (responder-derived) or allogeneic (stimulator-derived) PBMCs (*n* = 6 independent experiments). (**D**) Inhibition of proliferation by MAIT cells was analyzed separately in CD4^+^ and CD8^+^ responder T cells (*n* = 7 independent experiments). (**E**) Purified CFSE-labeled CD4^+^ T cells were cultured with allogeneic CD3^–^ PBMCs in the presence of purified MAIT cells or effector memory CD8 T cells (non-MAIT CD8^+^CD45RA^–^CCR7^–^ T_EM_ cells, CD8_EM_) at the indicated MAIT (or CD8_EM_)/CD4^+^ T cell ratios. Results are shown as mean ± SEM (**C**–**E**). *P 0.05, **P 0.01, ***P 0.001, ****P 0.001, from paired *t* tests (**A**) or 2-way ANOVA followed by Holm-Šídák multiple comparisons test (**D** and **E**).

We previously showed that MAIT cells do not proliferate in a mixed lymphocyte reaction (MLR) where CFSE-labeled responder PBMCs are cultured for 6 days with irradiated allogeneic stimulator PBMCs (26). PBMCs were randomly obtained from healthy donors from the national blood center, were not HLA-typed, and were thus considered as HLA mismatched. We used this MLR system to evaluate the regulatory function of MAIT cells and found that the proliferation of MAIT-depleted responder T cells was significantly increased as compared with that of unmanipulated T cells (which contained on average 3% of MAIT cells) (Figure 1A). In contrast, the addition of purified MAIT cells dose-dependently inhibited the proliferation of MAIT-depleted responder T cells, with about 40% average inhibition of proliferation at a 1:1 MAIT/responder T cell ratio and an inhibitory effect still detectable up to a 1:32 ratio (Figure 1, B and C). Importantly, this immunosuppressive effect of MAIT cells was observed regardless of their autologous or allogeneic origin (i.e., MAIT cells purified from the responder or stimulator population) (Figure 1C). Of note, the MAIT cell inhibitory effect was substantially more pronounced on CD4^+^ than on CD8^+^ responder T cells, for which the mean percentage inhibition remained below 20% even at the highest MAIT/responder T cell ratio (Figure 1D).

Human MAIT cells are mostly CD8^+^ and display an effector memory phenotype (12). To allow inclusion of a non-MAIT effector memory CD8^+^ T cell (CD8_EM_) population as a control in the MLR, we next used CFSE-labeled purified CD4^+^ T cells as responders and allogeneic CD3^–^ PBMCs as stimulators. The addition of MAIT cells, but not of purified CD45R^–^CCR7^–^ CD8_EM_ cells, strongly inhibited proliferation of alloreactive CD4^+^ T cells in a dose-dependent manner (Figure 1E), further validating an MAIT-mediated immunosuppressive effect. The proportion of annexin V^+^CD4^+^ T cells in the presence of MAIT cells remained negligible (less than 0.5%) throughout the culture, excluding a direct cytotoxic effect of MAIT cells on alloreactive CD4^+^ T cells (Supplemental Figure 2A; supplemental material available online with this article; https://doi.org/10.1172/jci.insight.166310DS1). As MAIT cells express CD25 upon activation, we then asked whether inhibition of CD4^+^ T cell proliferation might be related to competition for IL-2. However, the MAIT cell inhibitory effect was not modified when IL-2 (100 U/mL) was added during the MLR (Supplemental Figure 2B). It was also not mediated by the induction of regulatory T cells (Tregs) in the responder population as no change in the proportion of CD4^+^ Tregs was observed (Supplemental Figure 2C).

Taken together, our results indicate that MAIT cells exert a strong immunosuppressive effect on the proliferation of alloreactive CD4^+^ T cells.

### The inhibitory effect of MAIT cells on in vitro alloreactive T cell proliferation and activation is contact dependent and requires TCR-MR1 interaction.

**Figure 2:**
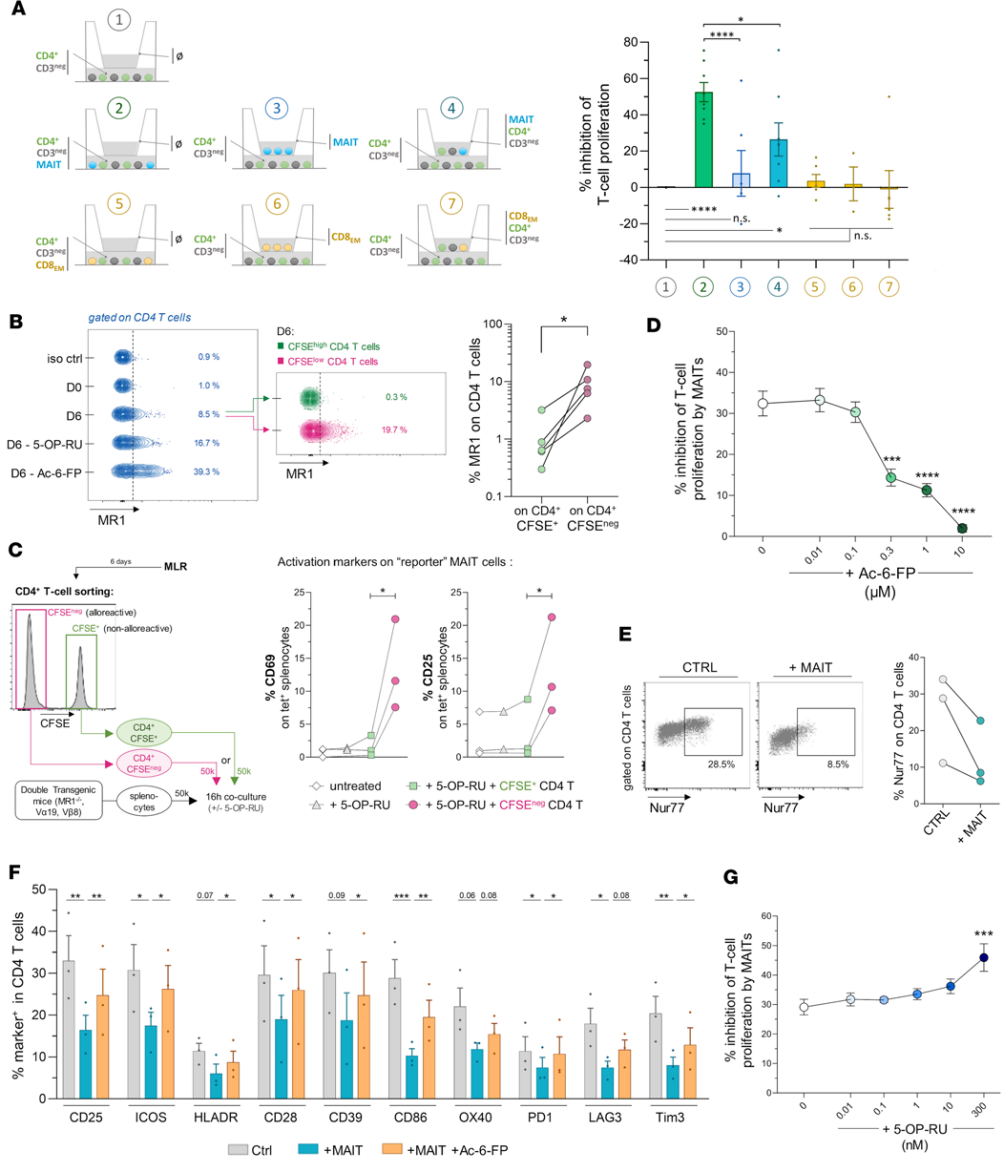
The inhibitory effect of MAIT cells on alloreactive T cells is partially contact dependent, requires TCR-MR1 interaction, and leads to inhibition of alloreactive T cell activation. (**A**) Responder CFSE-labeled CD4^+^ T cells with stimulator allogeneic CD3^–^ PBMCs, and MAIT cells (or CD8_EM_) at a 1:2 MAIT/CD4^+^ T cell ratio, were cultured in the upper and/or lower chamber of a Transwell plate, as indicated for each condition. Percentage (mean ± SEM) of inhibition of responder T cell proliferation (versus “condition 1” as reference) is shown for each culture setting. (**B**) Representative MR1 expression on responder CD4^+^ T cells at baseline (D0) and D6 of the MLR (where indicated, 5-OP-RU or Ac-6-FP was used to stabilize MR1 surface expression). (**C**) Human alloreactive (CFSE^–^) or nonalloreactive (CFSE^+^) human CD4^+^ T cells were sorted (at day 6 of the MLR) and cocultured with murine MAIT “reporter” cells obtained by isolating splenocytes from double-transgenic mice (Vα19 and Vβ8 TCR, Cα^–/–^, MR1^–/–^). After 16 hours in the absence or presence of 5-OP-RU (100 nM), expression of CD69 and CD25 was measured on murine MAIT reporter cells (MR1-tetramer^+^ T cells). (**D**) Percentage inhibition (mean ± SEM) of responder CD4^+^ T cell proliferation by MAIT cells (1:2 ratio) in the absence or presence of the inhibitory MR1 ligand Ac-6-FP at indicated concentrations. (**E**) Expression of Nur77 transcription factor in responder CD4^+^ T cells at D6 of the MLR. (**F**) Expression level (mean %, ± SEM) of indicated markers as assessed by spectral cytometry on responder CD4^+^ T cells on day 6 of the MLR in the absence (Ctrl) or presence of MAIT cells (1:2 ratio) alone or with Ac-6-FP (1 μM). (**G**) Percentage inhibition (mean ± SEM) of responder CD4^+^ T cell proliferation by MAIT cells (1:2 ratio) in the absence or presence of the activating MR1 ligand 5-OP-RU at indicated concentrations. Results are representative of at least of 3 independent experiments. *P 0.05, **P 0.01, ***P 0.001, ****P 0.001, from paired *t* tests or 1-way ANOVA followed by Tukey’s multiple comparisons test (**A**) or followed by Dunnett’s (**D**, **F**, and **G**).

To determine whether inhibition of alloreactive T cell proliferation by MAIT cells required direct cell contact, we physically separated MAIT cells from the responder and stimulator populations by a membrane insert (Transwell experiment). There was no longer any inhibition of alloreactive CD4^+^ T cell proliferation in the MLR (Figure 2A), suggesting that the MAIT cell immunosuppressive effect was cell contact dependent. Since MAIT cells alone in the upper chamber might not be in the same activation state as when added together with stimulator and responder cells, we also included a condition where MAIT cells were added in the upper chamber with the MLR occurring both in the upper and lower chamber (as depicted in Figure 2A, fourth condition). Interestingly, MAIT cells in this setting were able to inhibit proliferation of responder T cells in the lower chamber, albeit to a much lesser extent as compared with when MAIT cells were added in the lower chamber (Figure 2A). These data suggest that, although cell contact is required for inducing MAIT cell suppressive function, the actual suppressive effect may be — only partially — mediated by soluble factors.

Since MAIT cells were also able to inhibit anti-CD3/CD28–mediated CD4^+^ T cell proliferation (Supplemental Figure 3) (22), we thus inferred that MAIT cells probably interact directly with alloreactive CD4^+^ T cells and that their contact with antigen-presenting cells is not mandatory for their immunosuppressive effect.

Primary T cells show a very low membrane expression of MR1, which is markedly upregulated upon incubation with MR1 ligands (27). Of note, we found that MR1 was induced on CD4^+^ T cells during the MLR (in the absence of added ligands), almost exclusively on proliferating (CFSE^lo^) cells (Figure 2B), suggesting that a contact between MAIT cells and alloreactive CD4^+^ T cells may occur via the interaction between the MAIT cell TCR and MR1. Of note, in MLRs performed with total T cells as responder cells, we found lower expression of MR1 on CD8^+^ T cells as compared with CD4^+^ T cells (Supplemental Figure 4), which may be related to the lower MAIT-mediated suppressive effect observed on CD8^+^ T cells (Figure 1D).

As surface MR1 detection by flow cytometry is weak and challenging, probably because of transient surface expression and need for a stabilizing ligand (28), we aimed to functionally corroborate the presence of surface MR1 on allo-specific T cells by testing their ability to activate MAIT cells via the canonical MR1 ligand 5-OP-RU. To this purpose, we used murine MAIT “reporter” cells by isolating MAIT-enriched splenocytes from double-transgenic mice (Vα19 and Vβ8 TCR, Cα^–/–^, MR1^–/–^) (29, 30) in the presence of human allo-specific CD4^+^ T cells (CFSE^–^ cells sorted at day 6 of the MLR) or of control CD4^+^ T cells (CFSE^+^). In this setting, only human cells may present 5-OP-RU provided they express surface MR1 (mice splenocytes are MR1^KO^). After 16 hours of coculture, we found that MR1 tetramer^+^ MAIT “reporter” cells responded to 5-OP-RU (CD69/CD25 upregulation) in the presence of allo-specific CD4^+^ T cells (CFSE^–^) but not of “nonresponding” (CFSE^+^) CD4^+^ T cells (Figure 2C and Supplemental Figure 5). These data show that MR1-mediated activation of MAIT cells is possible through allo-specific CD4^+^ T cells.

To validate this hypothesis, we used Ac-6-FP, a potent inhibitory MR1 ligand that stabilizes expression of MR1 at the cell surface but prevents activation of the MAIT cell TCR (31). While Ac-6-FP increased expression of MR1 on CD4^+^ T cells (Figure 2B), it dose-dependently abrogated the immunosuppressive effect of MAIT cells on alloreactive T cell proliferation (Figure 2D). Importantly, Ac-6-FP alone had no effect on CD4^+^ T cell allo-proliferation in the MLRs (Supplemental Figure 6). These data suggest that TCR engagement on MAIT cells is required to elicit their suppressive function.

We then explored the impact of MAIT cells on the phenotypic characteristics of alloreactive CD4^+^ T cells. Expression of Nur77, a transcription factor that integrates signal strength downstream of the TCR within activated T cells (32), was decreased in CD4^+^ T cells (day 6 of the MLR) in the presence of MAIT cells (Figure 2E). Notably, the addition of MAIT cells in the MLR led to reduced surface expression of several activation-related markers (CD25, HLA-DR, PD-1, LAG3, Tim-3, CD39, OX40, CD28, ICOS, CD86) on responder CD4^+^ T cells (Figure 2F). These modifications were largely reversible in the presence of Ac-6-FP, verifying that the interaction between the MAIT cell TCR and MR1 is critical for MAIT suppression of CD4^+^ T cell activation in response to allogeneic signal.

Finally, we assessed whether MAIT cells maintained their immunosuppressive properties when exposed to the classical MR1-activating ligand, 5-OP-RU. Addition of 5-OP-RU stabilized expression of MR1 on CD4^+^ T cells (Figure 2B) and did not prevent MAIT-mediated inhibition of T cell alloreactivity, even at the highest dose (300 nM), where the inhibitory effect was further enhanced (Figure 2G), possibly reflecting higher MAIT proportion due to 5-OP-RU–induced proliferation (Supplemental Figure 7) or as a result of higher production of the responsible mediator.

Taken together, these results indicate that suppression of alloreactive CD4^+^ T cells by MAIT cells is contact dependent and involves recognition by the MAIT cell TCR of MR1, which is upregulated on alloreactive CD4^+^ T cells, leading to weaker TCR signaling and a consequent decrease in T cell activation and proliferation.

### Adoptive transfer of human MAIT cells protects from xeno-GVHD in immunodeficient mice.

To explore the immunosuppressive potential of human MAIT cells in vivo, we used a model of xenogeneic GVHD (xeno-GVHD) in which human PBMCs (huPBMCs) are injected into irradiated, immunodeficient NOD/SCID IL-2Rγ^null^ (NSG) mice. This leads to an aGVHD-like syndrome through expansion and extensive tissue infiltration of human T cells, resulting in severe tissue damage and death within 30–50 days (33–35). We recently demonstrated that MAIT cells contained in the transferred huPBMCs do not participate in immune-mediated tissue damage during xeno-GVHD (26).

**Figure 3:**
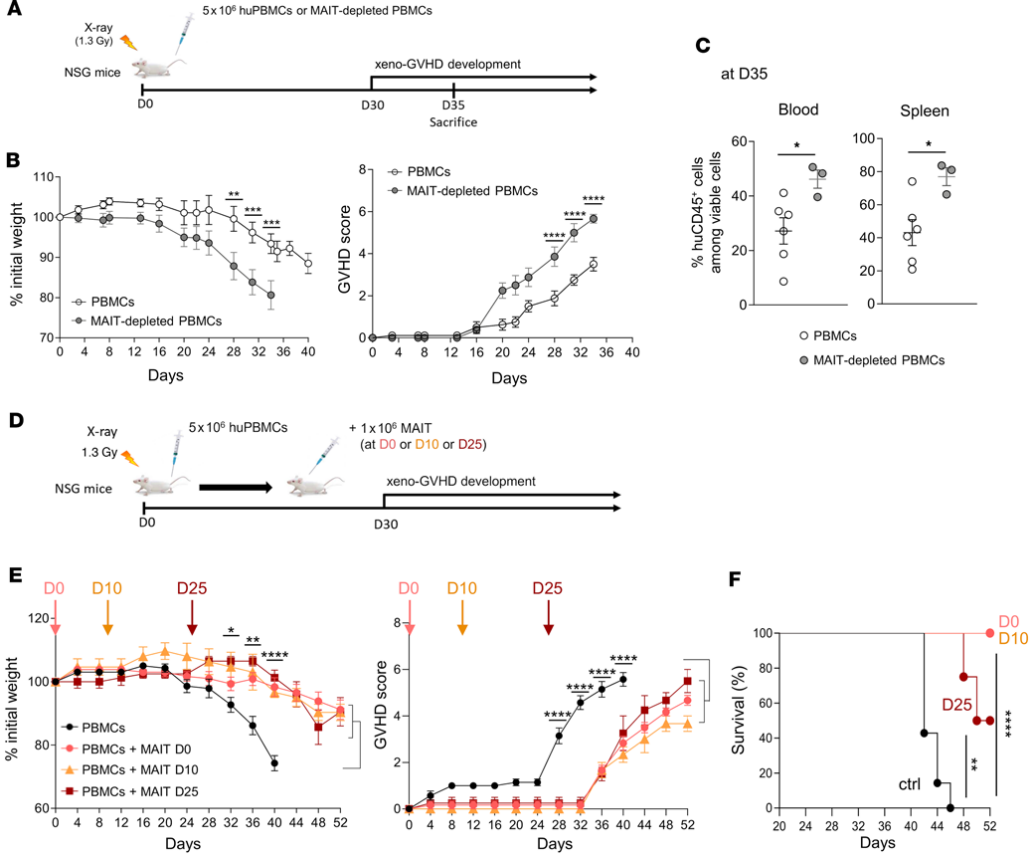
Adoptive transfer of human MAIT cells protects from xeno-GVHD in immunodeficient mice. (**A**) Irradiated (1.3 Gy) NSG mice were injected i.v. with 5 × 10^6^ human total PBMCs (huPBMCs) or MAIT-depleted PBMCs. (**B**) Weight loss and GVHD scoring were assessed from day 0 to sacrifice (day 35 or when the mice reached a GVHD score of 6) (*n* = 11 mice with weight data, and 8 with GVHD scoring, in the total PBMC group, and *n* = 8 mice in the MAIT-depleted PBMC group, from 3 independent experiments). (**C**) Frequencies of human CD45^+^ leukocytes among living cells in blood and spleen of mice sacrificed on day 35. (**D**–**F**) Irradiated NSG mice were injected with 5 × 10^6^ huPBMCs on day 0 without (*n* = 7, black), or with additional transfer of 1 × 10^6^ purified MAIT cells on day 0 (*n* = 6, red), day 10 (*n* = 3, orange), or day 25 (*n* = 4, brown). (**E**) Weight loss and GVHD scoring were assessed from day 0 to death (i.e., when GVHD score reached 6). Brackets indicate that statistical comparisons were performed between PBMCs and PBMCs + MAIT pooled groups. (**F**) Kaplan-Meier plots showing mouse survival in the indicated groups. Results are shown as mean ± SEM (**B**, **C**, and **E**). *P 0.05, **P 0.01, ***P 0.001, ****P 0.0001, from 2-way ANOVA followed by Holm-Šídák multiple comparisons test (PBMCs versus MAIT-depleted PBMCs in **B**, PBMCs versus PBMCs + MAIT pooled group in **E**), *t* tests (**C**), and log-rank tests (**F**).

Here, NSG mice were transferred with 5 × 10^6^ total or MAIT-depleted huPBMCs (donor-matched) and monitored to evaluate GVHD progression (Figure 3A). Of note, besides complete depletion in TCRVα7.2^+^ T cells, there were no substantial differences in the cellular composition between total and MAIT-depleted PBMCs (Supplemental Figure 8). Transfer of MAIT-depleted huPBMCs induced more severe GVHD than total huPBMCs, as evidenced by greater weight loss and higher disease score (hunching, activity, ruffling, and diarrhea) (Figure 3B). Mice were euthanized on day 35, mononuclear cells were isolated from peripheral blood and spleen, and the proportions of human CD45^+^ cells were determined. Consistent with more severe GVHD, mice injected with MAIT-depleted PBMCs showed a higher proportion of huCD45^+^ cells (almost exclusively consisting of T cells, as we reported; ref. 26), compared with those injected with total PBMCs (Figure 3C). These data suggest that MAIT cells constitutively present among transferred huPBMCs may limit the expansion of xenoreactive human T cells and delay xeno-GVHD.

We then performed adoptive transfer of 1 × 10^6^ purified MAIT cells, either at the same time as injection of huPBMCs (day 0) or later on (day 10 or day 25) (Figure 3D). To obtain enough cells, MAIT cells were short-term preamplified in vitro (Supplemental Figure 9A), a procedure that did not prevent their capacity to inhibit alloreactive T cell proliferation in the MLR (Supplemental Figure 9B). Adoptive transfer of MAIT cells at an early or delayed time point resulted in a striking reduction in weight loss and a delayed and less severe GVHD (Figure 3E). Moreover, mouse survival was significantly increased, especially when MAIT cells were transferred early (Figure 3F). These results indicate that MAIT cells can protect against the development of GVHD as well as control an ongoing GVHD.

### MAIT cells inhibit proliferation, activation, and effector function of xenoreactive T cells.

**Figure 4:**
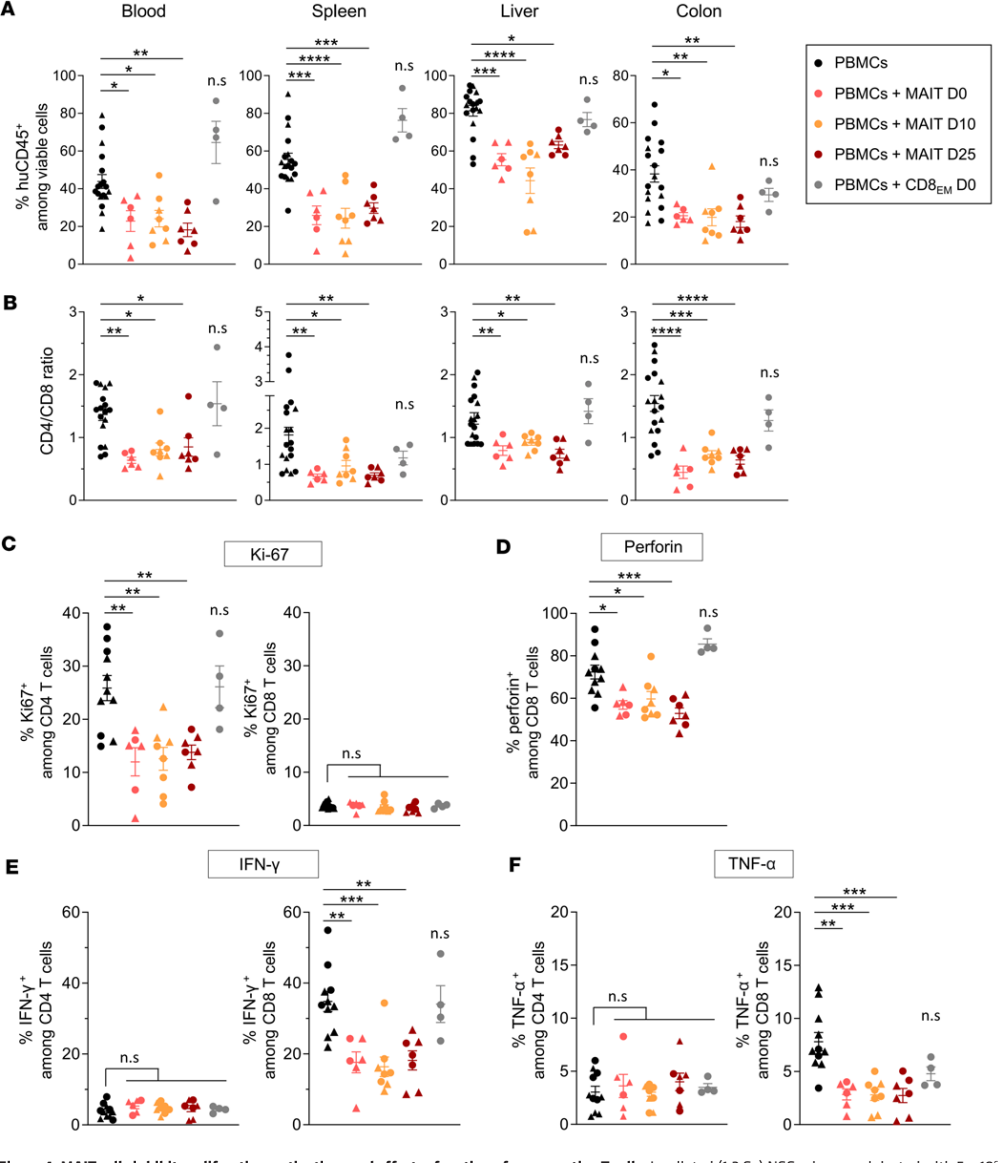
MAIT cells inhibit proliferation, activation, and effector function of xenoreactive T cells. Irradiated (1.3 Gy) NSG mice were injected with 5 × 10^6^ huPBMCs on day 0 without (*n* = 18, black) or with additional transfer of 1 × 10^6^ purified MAIT cells on day 0 (*n* = 6, red), day 10 (*n* = 8, orange), or day 25 (*n* = 7, brown), or of effector memory CD8^+^ T cells on day 0 (*n* = 4, CD8_EM_, gray). Mice were sacrificed on day 35, and cells from peripheral blood, spleen, liver, and colon were isolated. The proportion of human CD45^+^ leukocytes (huCD45^+^) among viable cells (**A**) and the CD4/CD8 T cell ratio (**B**) are shown in indicated compartments. (**C**) The proportion of intracellular Ki67^+^ cells (proliferating cells) in peripheral blood CD4^+^ and CD8^+^ T cells was determined by flow cytometry. (**D**–**F**) PBMCs were stimulated with phorbol 12-myristate 13-acetate (PMA)/ionomycin for 5 hours with brefeldin A/monensin added after 1 hour. Proportions of perforin^+^, IFN-γ^+^, and TNF-α^+^ cells among CD4^+^ and CD8^+^ T cells were determined by flow cytometry following intracellular staining. Results show individual values and mean ± SEM from 2 independent experiments (represented by circles or triangles). *P 0.05, **P 0.01, ***P 0.001, ****P 0.001, from 1-way ANOVA followed by Tukey’s multiple comparisons test.

To explore the effect of early or late transfer of MAIT cells on huPBMC engraftment, we analyzed human leukocytes infiltrating tissues in mice sacrificed on day 35. Regardless of transfer time, MAIT-treated mice showed significantly lower proportions and absolute numbers of huCD45^+^ cells in peripheral blood, spleen, liver, and colon compared with untreated or huCD8_EM_-treated mice (Figure 4A and Supplemental Figure 10A). MAIT cells were almost undetectable, regardless of whether they had been transferred early or late after huPBMCs (not shown), in agreement with our previous data showing that MAIT cells do not persist for long in NSG mice (26). Notably, while CD4^+^ T cells represented more than 60% of T cells (CD4/CD8 ratio 1) in all tissues of MAIT-untreated mice, their proportion strongly decreased in MAIT-treated mice, thus inverting the CD4/CD8 ratio (Figure 4B). Consistent with this observation, expression of the proliferation marker Ki-67 was markedly reduced in CD4^+^ T cells in MAIT-treated compared with untreated mice, whereas it was unchanged and low in CD8^+^ T cells (Figure 4C, spleen). Conversely, CD8^+^ T cells exhibited altered effector functions, as shown by the lower proportions of cells expressing perforin, IFN-γ, and TNF-α in MAIT-treated compared with untreated mice (Figure 4, D–F). IL-17 and IL-10 were not detectable in either population (data not shown).

**Figure 5:**
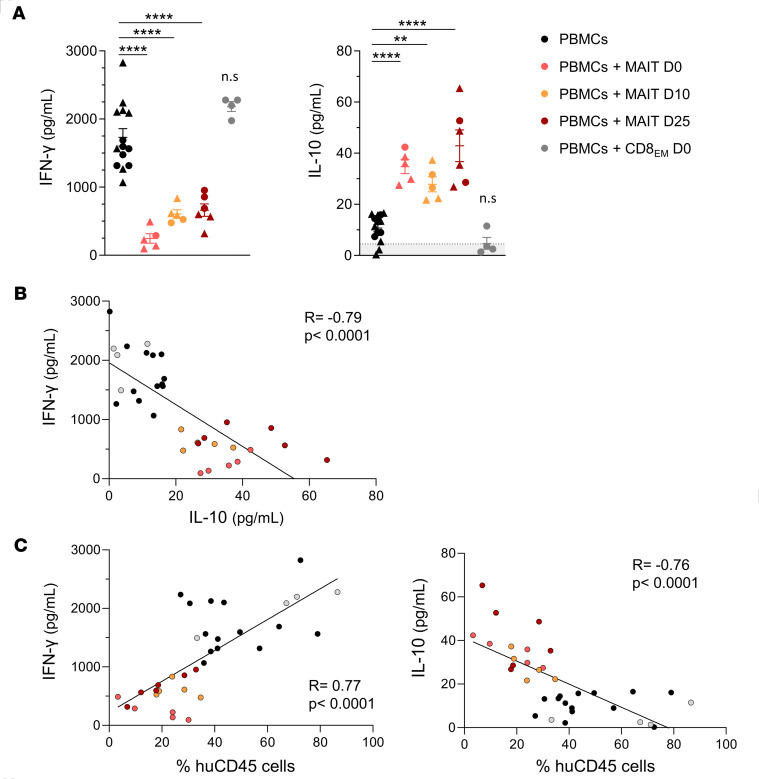
Circulating levels of IFN-γ and IL-10 correlate with MAIT cell–mediated control of xenoreactive T cell expansion. Irradiated (1.3 Gy) NSG mice were injected with 5 × 10^6^ huPBMCs on day 0 without (*n* = 14, black) or with additional transfer of 1 × 10^6^ purified MAIT cells on day 0 (*n* = 5, red), day 10 (*n* = 5, orange), or day 25 (*n* = 6, brown), or of effector memory CD8^+^ T cells on day 0 (*n* = 4, CD8_EM_, gray). Mice were sacrificed on day 35 and plasma was collected. (**A**) Circulating IFN-γ and IL-10 levels (mean ± SEM) were quantified by cytometric bead array in different groups; the shaded area indicates kit detection limit. **P 0.01, ****P 0.001, from 1-way ANOVA followed by Tukey’s multiple comparisons test. (**B**) Correlation between circulating IFN-γ and IL-10 levels. (**C**) Correlation of circulating IFN-γ or IL-10 levels with the proportion of huCD45^+^ cells in the peripheral blood. Spearman’s rank correlation coefficients (R) and corresponding *P* values are indicated.

Inflammatory cytokines were quantified in parallel in the serum. Consistent with the lower frequency of IFN-γ^+^ T cells, IFN-γ levels were much lower in MAIT-treated mice compared with control mice, while IL-10 levels were higher (Figure 5A) and negatively correlated with those of IFN-γ (Figure 5B). Importantly, the number of huCD45^+^ cells in peripheral blood correlated positively with circulating IFN-γ levels and negatively with those of IL-10 (Figure 5C). Levels of IL-2, IL-4, IL-6, TNF-α, and IL-17A were either undetectable or not different between treated or untreated mice (Supplemental Figure 10B).

Taken together, these results indicate that MAIT cells play an early immunoregulatory function during the development of GVHD via inhibition of CD4^+^ T cell proliferation and tissue infiltration, associated with inhibition of CD8^+^ T cell effector functions, and suggest that modulation of IFN-γ and IL-10 levels could participate in this effect.

### MAIT cells exert their immunosuppressive effect early after contact with huPBMCs via a TCR-MR1 interaction-dependent mechanism.

**Figure 6:**
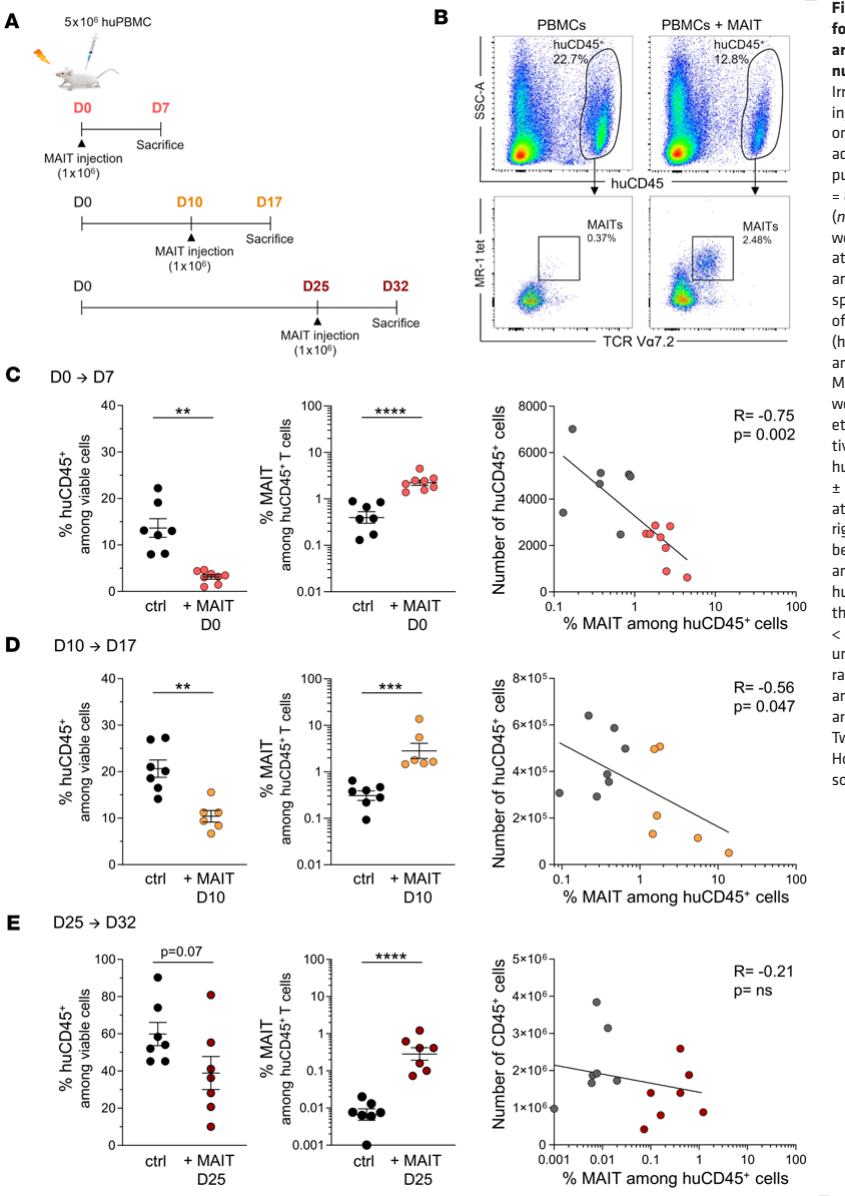
MAIT cells persist for at least 1 week in mice and are correlated with decreased numbers of huCD45^+^ cells. (**A**) Irradiated (1.3 Gy) NSG mice were injected with 5 × 10^6^ huPBMCs on day 0 without (*n* = 7) or with additional transfer of 1 × 10^6^ purified MAIT cells at day 0 (*n* = 8), day 10 (*n* = 6), or day 25 (*n* = 7). Mice were sacrificed 1 week after MAIT injection (i.e., at day 7, 17, or 32, respectively), and cells were recovered from spleen. (**B**) The proportions of human CD45^+^ leukocytes (huCD45^+^) among viable cells and of TCRVα7.2^+^ tetramer^+^ (tet) MAIT cells among huCD45^+^ cells were determined by flow cytometry, as shown on representative plots. (**C**–**E**) Proportion of huCD45^+^ and MAIT cells (mean ± SEM) is shown in each group at indicated time points, and right panels show correlations between MAIT cell frequencies and number of pathogenic huCD45^+^ leukocytes infiltrating the spleen. **P 0.01, ***P 0.001, ****P 0.001, from unpaired *t* tests. Spearman’s rank correlation coefficients (R) and the corresponding *P* values are indicated (right panels). Two-way ANOVA followed by Holm-Šídák multiple comparisons test.

The fact that MAIT cells were undetectable in blood and tissues of mice at the time of GVHD suggested that they exerted their immunosuppressive effect soon after their transfer. We therefore analyzed the content of huCD45^+^ cells and the presence of MAIT cells in mice sacrificed 1 week after early (day 0) or later (day 10 or 25) MAIT transfer (Figure 6, A and B). The proportions of huCD45^+^ cells in the spleen were already strongly reduced after early MAIT transfer compared with those in untreated mice (Figure 6C). Importantly, MAIT cells were easily detected 1 week after their transfer, and their proportion was inversely correlated to the number of huCD45^+^ cells (Figure 6C). Similar results were observed when MAIT cells were transferred later (day 10 or day 25) and mice sacrificed 1 week after (Figure 6, D and E), though the decrease in huCD45^+^ cells did not reach significance when MAIT cells were transferred on day 25, in line with the more limited improvement in mice survival in this setting. These data demonstrate an early, dose-dependent suppressive effect of MAIT cells on T cell proliferation in vivo, in agreement with the effect observed in vitro during the 6-day MLR.

**Figure 7:**
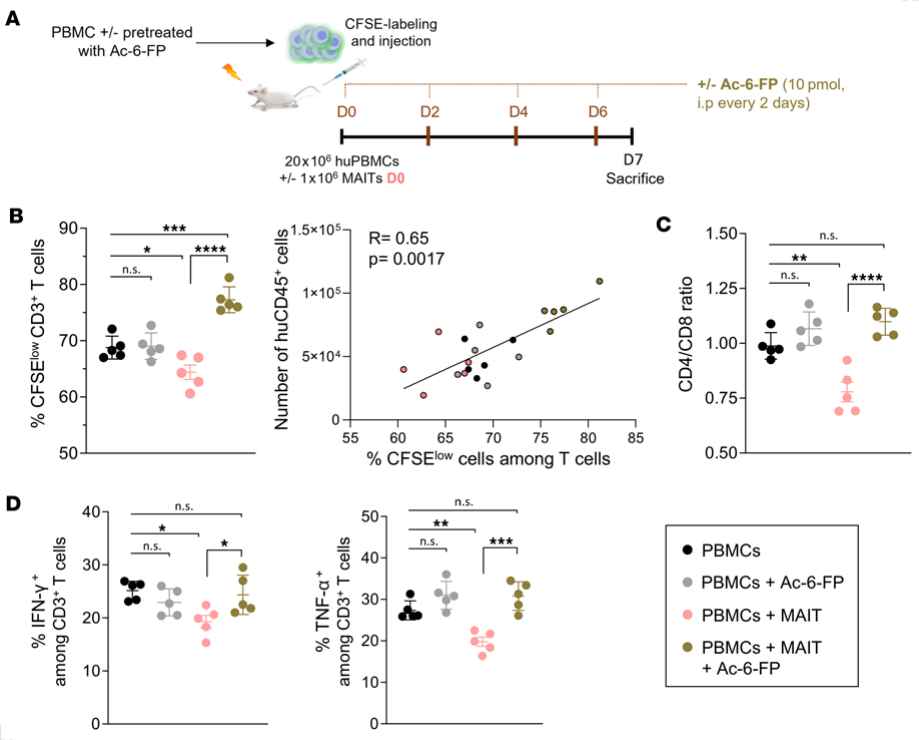
MAIT cell immunoregulatory function requires interaction of the TCR with MR1 in vivo. (**A**) Human PBMCs (20 × 10^6^) were pretreated or not with Ac-6-FP (10 μM) for 18 hours, CFSE-labeled, and injected in mice with or without cotransfer of 1 × 10^6^ purified MAIT cells. Ac-6-FP (10 pmol) was injected intraperitoneally on D0, 2, 4, and 6 in mice that had or had not (as control) received MAIT transfer. Mice were sacrificed on D7 and cells were recovered from the spleen (*n* = 5 for each condition). (**B**) Percentage of proliferating (CFSE^lo^) T cells (mean ± SEM) and correlation with the number of huCD45^+^ cells. Spearman’s rank correlation coefficient (R) and the corresponding *P* value are indicated. (**C**) CD4/CD8 T cell ratio in the different groups. (**D**) Splenic cells were stimulated with PMA/ionomycin for 5 hours, with brefeldin A/monensin added after 1 hour. Proportions of IFN-γ^+^ and TNF-α^+^ cells among T cells were determined by flow cytometry following intracellular staining. *P 0.05, **P 0.01, ***P 0.001, ****P 0.0001, from 1-way ANOVA followed by Tukey’s multiple comparisons test.

To determine whether this suppressive effect of MAIT cells required direct cell contact with T cells via a TCR-MR1 interaction, mice were cotransferred on day 0 with MAIT cells and with CFSE-labeled huPBMCs that had been pretreated with the antagonist MR1 ligand Ac-6-FP and were also given Ac-6-FP i.p. every 2 days before sacrifice on day 7 (Figure 7A). T cell proliferation was quantified in the spleen according to CFSE dilution. As expected, in the absence of Ac-6-FP treatment, the proportions of CFSE^lo^ proliferating T cells were lower in MAIT-treated mice than in untreated mice and correlated with total huCD45^+^ cell counts (Figure 7B). This proliferation inhibition primarily affected CD4^+^ T cells, as reflected by lower CD4/CD8 T cell ratios (Figure 7C). In addition, T cell effector functions were already impaired, as shown by the lower expression of IFN-γ and TNF-α (Figure 7D). Remarkably, all these effects were abrogated by Ac-6-FP treatment (Figure 7, B–D).

Together, these data demonstrate that MAIT cells dose-dependently inhibit T cell proliferation, activation, and effector functions after their transfer, and this effect requires an early interaction of their TCR with MR1.

## Discussion

Recent studies have demonstrated both inflammatory and regulatory functions for MAIT cells in various mouse models and human diseases (36–39). In the HCT setting, studies from our group and others have linked MAIT cells with lower rates of acute and chronic GVHD (17, 21–26, 40), and some of these studies were able to link posttransplant MAIT cell reconstitution with the intestinal microbiota (17, 22–24, 40). In preclinical models, conditioning-resistant host residual MAIT cells were protective against acute gut GVHD via IL-17–mediated control of microbial translocation, thus attenuating pathogenic proinflammatory T cell responses (17). In humans, a recent study nicely demonstrated the relationship between high fecal microbial diversity, MAIT cell count, and favorable post-HSCT outcome and showed that MAIT cells undergo transcriptional changes consistent with a gain in cytotoxic and effector functions that may reflect their role in controlling bacteria or pathogenic cell populations (24).

Here, we identify human MAIT cells as important immunoregulatory cells capable of directly suppressing alloreactive T cell proliferation and activation in a classical in vitro model of alloreactivity. Using a preclinical model of xeno-GVHD induced by host-reactive human T cells, we further demonstrate that MAIT cells greatly delay GVHD onset and severity after their early adoptive transfer. Moreover, MAIT cells are also able to control ongoing GVHD when transferred at delayed time points. This effect is associated with a striking decrease in the number, proliferation, and effector function of human T cells infiltrating tissues of diseased mice and is correlated with lower circulating levels of IFN-γ and TNF-α and increased IL-10 levels.

In contrast with previous studies, this immunosuppressive capacity of MAIT cells is likely microbially independent, since bacterial metabolites were absent from the in vitro model and absent — or present in very low amounts — in NSG mice bred under specific pathogen–free conditions. Still, it cannot be ruled out that microbially derived MAIT antigens may be present in specific pathogen–free mice and could play a role in the process. Importantly, however, the suppressive function of MAIT cells was preserved in the presence of high amounts of the classical microbially derived ligand, 5-OP-RU, suggesting that it will occur even under (pathological) conditions where bacteria are present. MAIT cells did not suppress host-reactive T cells through direct killing, in contrast with other populations of Tregs (such as CD4^+^ and CD8^+^ Tregs and KIR^+^CD8^+^ T cells) (41–43). They did not express Foxp3 (data not shown), thus distinguishing them from the recently described 5-OP-RU–induced MAIT cell subset that phenotypically resembled conventional Tregs (44). Finally, in contrast to NKT cells (45), their suppression of GVHD was unlikely to be related to IL-4 production, as suggested by undetectable circulating levels of this cytokine in both MAIT-treated and untreated mice. However, rapid consumption of IL-4 in tissue may prevent its detection such that a role for IL-4 cannot be definitely ruled out.

We show that MAIT cells act in a cell contact–dependent manner, which requires an early and direct interaction of their TCR with MR1 induced on host-reactive T cells, as shown by the dose-dependent reversal of the MAIT cell immunosuppressive effect by the MR1-inhibitory ligand Ac-6-FP. Importantly, MR1 is mostly intracellular at steady state, and ligand availability seems to be the limiting factor for its cell surface translocation (46). Therefore, a key question concerns the nature of the ligand allowing MR1 expression in host-reactive T cells, and whether it is supplied endogenously or is obtained from the extracellular environment in this setting. MR1 can present ligands distinct from riboflavin precursors and folic acid–derived metabolites, such as undefined endogenous ligand(s) (47) and organic compounds with diverse chemical scaffolds, which are able to modulate the activity of MAIT cells (48). Such ligand(s) could deliver a regulatory signal to MAIT cells to suppress the activation and effector functions of host-reactive T cells. Alternatively, it is possible that the use of different TCRβ chains by specific subsets of MAIT cells is able to fine-tune the responsiveness to certain MR1 ligands. In any case, such a mechanism might also play a crucial role in homeostasis to control activation of MAIT cell effector functions in the absence of microbial infection.

Whether MAIT suppressive activity is exclusively TCR-MR1 dependent or also relies on other receptor-ligand interactions or soluble factors remains unclear. Tregs have been recognized to impart immunosuppressive effect through contact-dependent (e.g., CTLA-4, LAG3) and -independent mechanisms (release of IL-10, TGF-β) (49). We found increased circulating levels of IL-10 in MAIT-treated mice, which negatively correlated with IFN-γ levels and with the number of human T cells infiltrating tissues, but we could not determine the source of IL-10 in our models. Whether increased IL-10 is the cause or consequence of decreased IFN-γ is unclear, as IFN-γ inhibits IL-10 production, but IL-10 also limits activation and differentiation of effector T cells. In murine allogeneic HCT models, IL-10 increase (following ST2 blockade) was associated with reduced expansion of pathogenic T cells and production of inflammatory cytokines, such as IFN-γ (50).

We did not determine the potential contribution of MAIT tissue repair functions, but this may be an additional mechanism at work in the control of GVHD. Previous work suggested that MAIT-deficient mice had impaired intestinal barrier integrity after HCT (17). MAIT cells can produce IL-22, a cytokine involved in epithelial barrier protection, though it is also endowed with inflammatory function depending on the inflammatory context (51). MAIT cells can also produce high levels of amphiregulin (16), a ligand for the epidermal growth factor receptor expressed mainly on epithelial cells and stem cells. These data indicate that MAIT cells can play a complex, multifaceted role in maintaining immune homeostasis and promoting transplantation tolerance.

One of the biggest hurdles to the development of a successful GVHD therapy is the preservation of the therapeutic GVL effect to avoid relapse in allo-HCT recipients. MAIT cells have been reported to exert antitumor functions in several studies, though pro-tumor roles have been described in some cases (52, 53). While previous clinical studies in HSCT recipients have linked higher early MAIT cell frequency with less aGVHD, decreased transplantation-related mortality, and prolonged overall survival, none of them reported an effect on malignant disease progression or relapse. We did not examine whether MAIT cells impact the clearance of tumor cells by conventional T cells in our preclinical model. Importantly, however, we recently showed that MAIT cell frequencies 1 year after allo-HCT were significantly increased in patients with long-term remission of acute myeloid leukemia as compared with those with subsequent relapse (54). Taken together, these data suggest that MAIT cells preserve donor antileukemia activity, a key benefit of allo-HCT.

From a translational perspective, the ability of MAIT cells to suppress autologous and allogeneic (third-party) T lymphocytes makes them attractive candidates for universal immune adoptive strategies aimed at regulating GVHD in the clinic, with no practical need for individualized MAIT cell production for each patient. Moreover, in vitro short-term expanded MAIT cells retain their regulatory functions, compared with freshly isolated and directly infused MAIT cells, which is crucial for the feasibility of off-the-shelf adoptive cell therapy. MAIT cells offer the advantage that they come from a readily available pool of circulating T cells and are thus amenable to efficient clinical-scale production. They are abundant in the peripheral blood from which they can be easily purified and manufactured under Good Manufacturing Practice. Moreover, due to their expression of the multidrug efflux protein ABCB1 (12), MAIT cells are relatively resistant to immunosuppressive drugs, such as cyclosporin A used for GVHD prevention or treatment. Importantly, we show that MAIT cells exert their regulatory effect very soon after their adoptive transfer, and this effect is maintained while they become hardly detectable more than 1 week after transfer, meaning that there is no need for their persistence. Thus, MAIT cell adoptive therapy might be highly beneficial in the context of allo-HCT whereby GVHD suppression and GVL maintenance could both be achieved.

Our study has some limitations. First, the precise mechanisms used by MAIT cells to suppress GVHD remain incompletely understood, though we show that they result in considerable suppression of host-reactive T cell proliferation and production of the proinflammatory cytokines IFN-γ and TNF-α, as observed for Tregs (55). In addition to a direct contact between the MAIT cell TCR and MR1 leading to inhibition of early TCR signaling in host-reactive T cells, distinct mechanisms may be at work, such as induction of IL-10 production by so far unidentified cells or suppression mediated by coinhibitory receptors such as LAG3. Whether the requirement for an interaction between the MAIT cell TCR and MR1 in vivo involved cells other than host-reactive human T cells cannot not be excluded given the strong MR1 conservation between species. Besides, the kind and extent of suppression may depend on the timing, intensity, and duration of GVHD and on the anatomical sites involved.

Second, although we used relevant in vitro and preclinical models that supported previous clinical studies, for obvious reasons it was not possible to show directly in human tissue samples that MAIT cells prevent the proliferation and effector functions of alloreactive T cells. Finally, a critical point will be to determine whether regulatory functions of MAIT cells are stable under inflammatory conditions, or whether a particular MAIT subset with a given signature is predominant, depending on the environment and the availability of MR1 ligands.

In conclusion, we demonstrate here a previously unrecognized, microbially independent, immunosuppressive function of human MAIT cells. Notably, the immunosuppressive role of MAIT cells may be more general and most probably extends to other kinds of T cell specificities beyond alloreactivity. In addition to providing useful information to understand the key cellular dynamics of GVHD, our study could pave the way for new therapeutic approaches to control GVHD and possibly other inflammatory disorders beyond this context.

## Methods

### Study design.

The objective of this study was to investigate the immunoregulatory function of human MAIT cells. To this end, we used an in vitro model of MLR and a preclinical model of xenogeneic GVHD in immunodeficient mice after transfer of human PBMCs. All experiments with human cells and animals were performed in strict accordance with French ethics laws under approved procedures (as described in *Study approval*).

### Human biological samples and processing.

Healthy adult blood was collected from residual leukocyte packs after blood donation (Etablissement Français du Sang). PBMCs were isolated by density gradient centrifugation (Ficoll-Paque) within 2–3 hours after blood sampling and cryopreserved in heat-inactivated fetal calf serum–containing medium with 10% dimethyl sulfoxide (DMSO). PBMCs were thawed at least 4 hours and no more than 12 hours before use.

In all in vitro experiments, cells were cultured in RPMI-1640 (Invitrogen, Thermo Fisher Scientific) containing 10% human AB serum (EuroBio) and 1% penicillin/streptomycin (Invitrogen, Thermo Fisher Scientific).

### Purification and expansion of MAIT cells.

MAIT cells, defined as CD3^+^ MR1:5-OP-RU-tetramer^+^ TCR Vα7.2^+^ lymphocytes, were sorted using a FACSAria III cell sorter (BD Biosciences). The gating strategy of MAIT cells is shown in Supplemental Figure 1. CD8_EM_ cells, defined as CD3^+^CD8^+^CD45RA^–^CCR7^–^ cells, were sorted in parallel from the same donor as controls. Viability dye (FVS700, BD Biosciences) was used to exclude dead cells. Sorted cells were used immediately after counting, either for in vitro experiments or in vivo adoptive transfers.

Where indicated, MAIT cells were expanded for 6 days from total PBMCs in human T cell culture medium supplemented with IL-2 (100 U/mL, Miltenyi Biotec) and 300 nM 5-OP-RU synthesized as described in (56–58).

### In vitro T cell proliferation assays.

MLRs were performed using CFSE-labeled PBMCs (responders, 1 × 10^6^/mL) incubated with γ-irradiated (40 Gy) allogeneic PBMCs (stimulators, 1:1 ratio) in 96-well, U-bottom plates. CFSE labeling (5 μM) was performed using CellTrace CFSE Cell Proliferation Kit (Thermo Fisher Scientific) according to the manufacturer’s instructions. Alternatively, CD4^+^ T cells (responders) were purified from PBMCs using indirect magnetic labeling (REAlease CD4 MicroBead Kit, Miltenyi Biotec), and allogeneic CD3^–^ PBMCs (stimulators) were obtained using T cell depletion with the CD3 MicroBeads kit (Miltenyi Biotec) according to manufacturer’s instructions. CFSE-labeled CD4^+^ T cells (2 × 10^6^/mL) were cocultured with allogeneic stimulator CD3^–^ cells (1 × 10^6^/mL) in 96-well, flat-bottom plates for 6 days. MAIT cells or effector memory (non-MAIT) CD8^+^ T cells were FACS-sorted as described above and added (on day 0) to the MLR at the indicated ratios to CFSE-labeled responding cells. When indicated, IL-2 (100 U/mL, Miltenyi Biotec), 5-OP-RU, or Ac-6-FP (Cayman Chemical Company) was added to the MLR (on day 0) at indicated concentrations. Transwell experiments were performed using a 96-well Transwell plate (Corning) in which responder CD4^+^ T cells were cocultured with allogeneic CD3^–^ cells in the lower (or both lower and upper) chamber, and MAIT cells (or control memory CD8^+^ T cells) were added either in the lower or upper chamber. Cells were harvested on day 6 for T cell proliferation analysis by flow cytometry.

For anti-CD3/CD28–mediated T cell stimulation experiments, CD3^+^ T cells were sorted using the CD3 MicroBeads kit (Miltenyi Biotec), CFSE-labeled, and cultured (2.5 × 10^5^/mL) in the presence of Dynabeads Human T-Activator CD3/CD28 (Thermo Fisher Scientific) for 5 hours (cell-to-bead ratio 1:1). The beads were then magnetically removed and MAIT cells/memory CD8^+^ T cells were added to the culture at indicated ratios. Cells were harvested on day 4 for T cell proliferation analysis by flow cytometry.

### Flow cytometry.

Cells were harvested from in vitro experiments or isolated from mouse organs (as described below) (26) and stained for surface markers for 20 minutes at 4°C in staining buffer (PBS with 0.5% BSA and 0.01% sodium azide). To identify MAIT cells, we used the specific MR1:5-OP-RU tetramer (provided by the NIH Tetramer Core Facility) for 45 minutes at room temperature prior to surface staining with anti-CD3 and anti-TCRVα7.2 antibodies. All antibodies used in the study are listed in Supplemental Tables 1 and 2. For MR1 staining, an isotype control staining was used to set the gates on the corresponding subpopulation. Amine-reactive dyes Fixable Viability Stain 700 (FVS700, BD Biosciences) or SYTOX Blue (Thermo Fisher Scientific) were used according to the manufacturer’s instructions to exclude dead cells in all analyses. For the evaluation of intracellular antigens, cells were then fixed (2% paraformaldehyde [PFA] for 30 minutes at 4°C), washed, and stained with antibodies targeting intracellular markers in permeabilization buffer (PBS with 0.5% BSA and 0.2% saponin) for 20 minutes at 4°C.

For intracellular cytokine assays, fresh single-cell suspensions from spleen, liver, and colon of the indicated groups were incubated in a 48-well, flat-bottom plate for 5 hours with the eBioscience Cell Stimulation Cocktail (PMA 80 ng/mL and ionomycin 1.3 μM, Thermo Fisher Scientific). The eBioscience (Thermo Fisher Scientific) Protein Transport Inhibitor Cocktail (brefeldin A 10 μM and monensin 2 μM, Thermo Fisher Scientific) was added for the last 4 hours of incubation. Cells were harvested and stained for surface markers, before fixation (2% PFA), permeabilization, and intracellular staining.

Multiparametric flow cytometry analyses were performed using a Celesta cytometer (BD Biosciences). Spectral flow cytometry analyses were performed using an Aurora cytometer (Cytek Biosciences). Data were analyzed using FlowJo v10.6.1 software and the OMIQ and Cytobank online platforms.

### Animals.

NSG mice (Jackson Laboratory) were bred in the animal facility of Saint Louis Research Institute and housed under specific pathogen–free conditions. Eight- to 10-week-old female mice were used in adoptive transfer experiments. All appropriate procedures were performed in the animal facility (registration number B75-10-08) and followed to ensure animal welfare.

### Xeno-GVHD.

Mice were irradiated (1.3 Gy) 24 hours prior to intravenous injection of 5 × 10^6^ human PBMCs in the caudal vein. In some experiments, PBMCs from the same donors were depleted of MAIT cells before intravenous injection. To this end, PBMCs were stained with biotin-labeled anti-TCRVα7.2 antibody (BioLegend) followed by incubation with streptavidin MicroBeads (Miltenyi Biotec) to deplete TCRVα7.2^+^ cells (containing MAIT cells) by magnetic cell separation. Mice were monitored daily and GVHD development was scored 3 times per week, based on weight loss, hunching posture, reduced mobility, and hair loss, as described (59). A total of 1 × 10^6^ ex vivo purified or short-term expanded MAIT cells (as described above) were injected in the orbital sinus of mice on the indicated days (i.e., the day of PBMCs injection [D0], D10, or D25 after PBMCs’ injection). CD8_EM_ cells as defined above served as controls (1 × 10^6^ cells, retro-orbital injection). Peripheral blood was harvested at indicated time points from the subclavian vein for flow cytometry analysis, and serum was collected and frozen (for measurement of cytokine levels). At specific times, or when mice reached a GVHD score greater than 6 (59), mice were sacrificed by cervical dislocation. Organs (spleen, liver, colon, lungs, and bone marrow) were collected in PBS 1×–BSA 1% solution and immediately processed as described below and analyzed by flow cytometry.

### Isolation of cells from mouse tissues.

Briefly, blood, spleen, and liver were prepared as single-cell suspensions. Colon was placed in RPMI-1640 medium and digested with collagenase VIII and DNase I. Mononuclear cells from liver and colon were isolated by Percoll density gradient centrifugation. Red blood cells from the blood and spleen were lysed using FACS lysis solution (BD Biosciences) and ACK solution (Thermo Fisher Scientific), respectively.

### Quantification of circulating cytokines.

Serum was collected at the indicated time points, and cytokine levels (TNF-α, IFN-γ, IL-2, IL-4, IL-6, IL-10, and IL-17A) were determined using the Cytometric Bead Array Th1/Th2/Th17 kit (BD Biosciences) according to the manufacturer’s instructions.

### In vivo blocking of the TCR-MR1 interaction.

NSG mice were irradiated (1.3 Gy) 24 hours prior to intravenous injection of 20 × 10^6^ human PBMCs previously labeled with CFSE (5 μM, Thermo Fisher Scientific) in the caudal vein, with or without retro-orbital injection of 1 × 10^6^ MAIT cells. PBMCs were pretreated for 18 hours with Ac-6-FP (10 μM) or DMSO as control before injection, and Ac-6-FP (10 pmol/mouse) was injected i.p. on days 0, 2, 4, and 6 after PBMCs’ injection. Mice were sacrificed on day 7, and the spleen was harvested and processed before cell analysis by flow cytometry.

### Sex as a biological variable.

Sex was not considered as a biological variable in this study as xenogeneic GVHD development was similarly shown in male and female mice (33). Our study exclusively examined female mice because they can share the same space without rivalry and for animal well-being (absence of fighting).

### Statistics.

Comparisons between in vitro conditions (within same individuals) or between mouse groups were evaluated using the 2-tailed paired or unpaired *t* test, respectively (directly or after log transformation). Associations between variables were assessed using the Spearman’s rank correlation test. One-way or 2-way ANOVA followed by Tukey’s or Holm-Šídák multiple comparisons tests, respectively, were used where indicated. *P* values less than 0.05 were considered significant. The Kaplan-Meier approach was used to assess mouse survival, and the log-rank test was performed to compare survival rates between groups. All statistical analyses were performed using Prism version 8 (GraphPad Software).

### Study approval.

A written agreement was obtained from each healthy blood donor to use the cells for research purposes, in accordance with French ethics laws and with the approval of Etablissement Français du Sang, Paris, France. All animal experiments were performed in strict accordance with Institutional Animal Care and Use Ethics Committee under approved procedures (Ministère de l’Enseignement Supérieur et de la Recherche and Comité d’éthique Paris-Nord, Paris, France, 2018090511164693.apafis #16624).

### Data availability.

Data behind all reported means are available in the Supporting Data Values XLS file.

## Author contributions

NTB, MFC, GS, OL, and SCZ were responsible for conceptualization. NTB, MFC, MS, VP, OL, and SCZ developed methodology. NTB, ML, NM, MT, AB, VP, TY, and IM performed investigation. OL and SCZ acquired funding. MFC, GS, and SCZ supervised. NTB, MFC, and SCZ wrote the original draft. NTB, MFC, GS, OL, and SCZ reviewed and edited the manuscript. NTB, ML, and MFC made equally important contributions, and first authorship order was based on the amount of work each author contributed to the study.

## Supplementary Material

Supplemental data

Supporting data values

## Version 1 02/01/2024

In-Press Preview

## Version 2 03/08/2024

Electronic publication
