# Supplementary material for: Human MAIT cells inhibit alloreactive T cell responses and protect against acute graft-versus-host disease
Source: JCI Insight. 2024 Feb 1;9(5):e166310. doi: 10.1172/jci.insight.166310 (PMC11143928; doi:10.1172/jci.insight.166310)
Supplement: Supplemental data [file jciinsight-9-166310-s008.pdf]

SUPPLEMENTARY MATERIAL

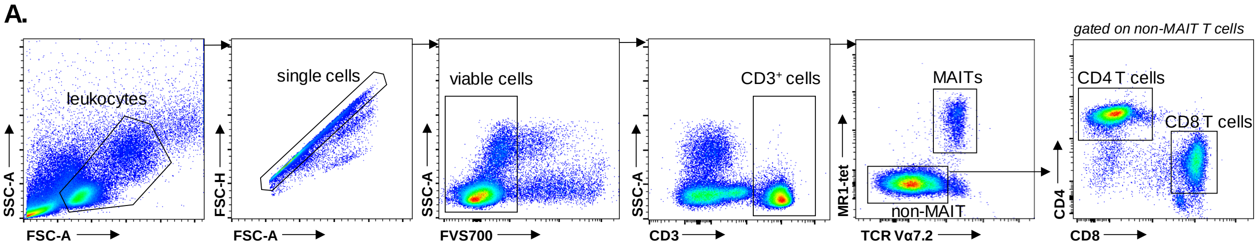

Suppl Fig. 1: Gating strategy for MAIT cells identification and sorting.

Cells were stained with (at least) anti-CD3, anti-CD4, anti-CD8, anti-TCR-Vα7.2 antibodies, MR1-tetramers and a viability dye (FVS700), and the gating strategy is indicated with debris and doublets exclusion, selection of live (FVS700^{neg}) cells, total T cells (CD3^{+}) and MAIT (TCR-Vα7.2^{+}tetramer^{+}) or non-MAIT (TCR-Vα7.2^{neg}tetramer^{neg}) cells.

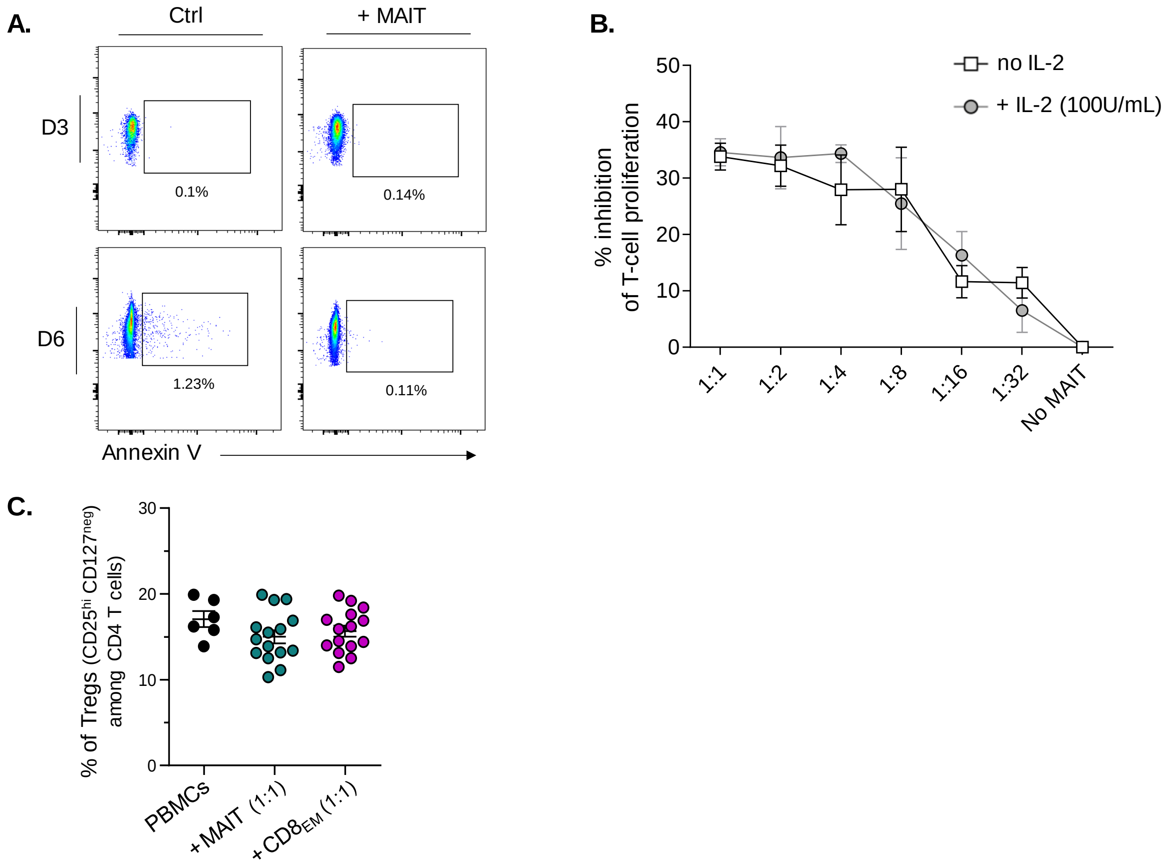

Suppl Fig. 2: MAITs suppressive function did not rely on cytotoxicity toward responder T cells, IL-2 deprivation, nor expansion of Tregs during the MLR.

Responder CFSE-labeled CD4 T cells were cultured with stimulator allogeneic CD3-negative PBMCs in the absence or presence of MAITs (1:2 MAIT:CD4 T-cell ratio). (**A**) Annexin-V staining on CD4 T cells in the absence (Ctrl) or presence of MAIT on day 3 and day 6 of the MLR. (**B**) Percentage inhibition (mean ± SEM, n=4) of responder CD4 T cell proliferation by MAITs in the absence or presence of IL-2 (100U/mL) during the MLR. (**C**) Percentage of Tregs (defined as CD25^{hi}CD127^{neg}) among CD4 T cells on day 6 of the MLR in the absence or presence of MAIT or of CD8 T_{EM} cells.

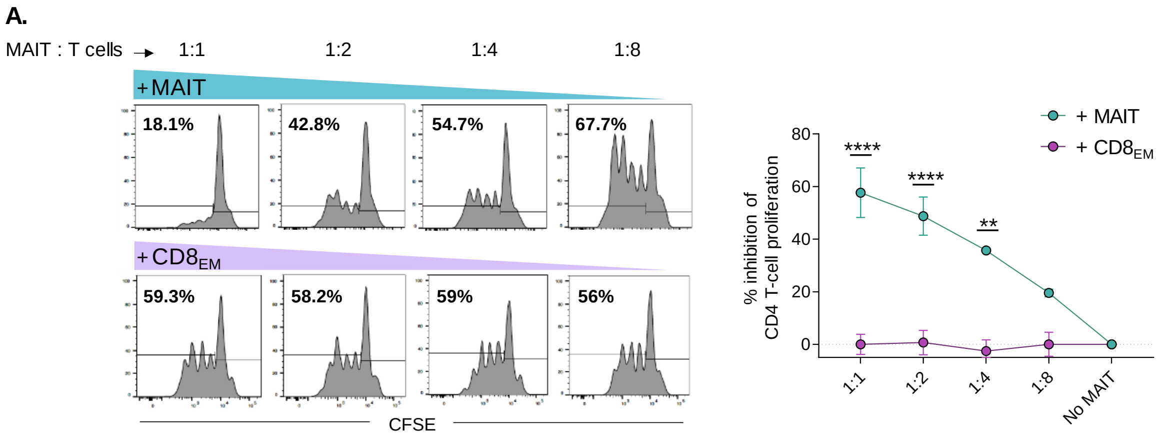

<u>Suppl Fig. 3</u>: MAITs dose-dependently inhibit the proliferation of anti-CD3/CD28-stimulated T cells.

CFSE-labeled T cells were stimulated for 5h with anti-CD3/anti-CD28 microbeads (1:1 cell-to-bead ratio). Beads were then magnetically removed and MAITs or effector memory CD8 T cells ($CD8_{EM}$) were added to the culture at indicated ratios. Cells were harvested on day 4 for T-cell proliferation analysis by flow cytometry. Left: Representative example showing CFSE staining in non-MAIT CD4 T cells; percentage of cells with at least one division indicated on each plot. Right: Summary graph showing percentage of inhibition of CD4 T cell proliferation (mean ± SEM, 3 independent experiments) by MAITs (or $CD8_{EM}$) at the indicated MAIT (or $CD8_{EM}$): CD4 T-cell ratios. **p<0.01, ****p<0.001, from two-way ANOVA followed by Sidak's multiple comparisons test

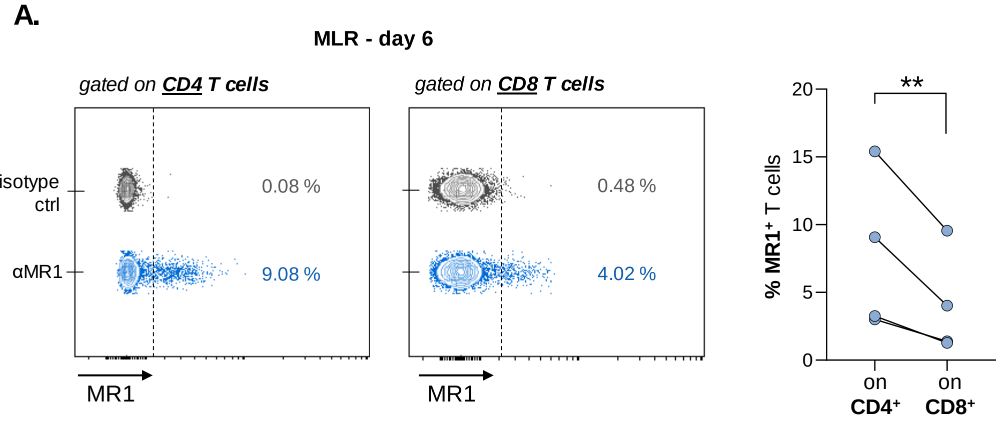

<u>Suppl Fig. 4</u>: Comparison of MR1 expression on CD4 and CD8 T cells.

Responder CFSE-labeled total ($CD3^{+}$) T cells were cultured with stimulator allogeneic CD3-negative PBMCs and MR1 staining was performed at day 6 of the MLR. **p<0.01, from paired t-tests, in 4 independent experiments.

A. Activation markers on "reporter" MAIT cells :

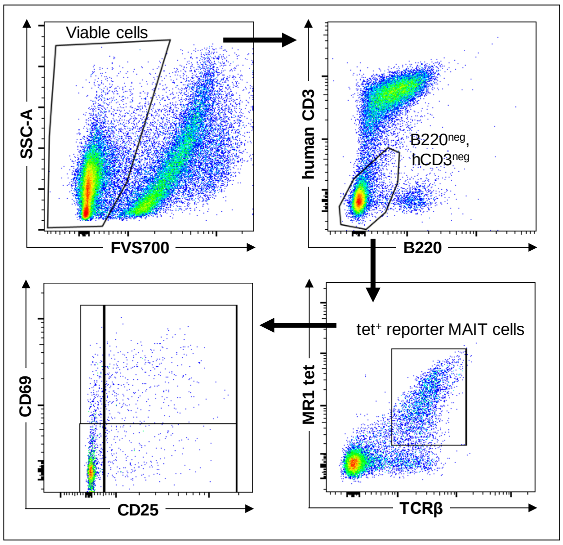

Suppl Fig. 5: Alloreactive CD4 T cells can activate MAIT cells via an MR1-restricted ligand

(related to Fig.2C)

Responder CFSE-labeled CD4 T cells were cultured with stimulator allogeneic CD3-negative PBMCs. At day 6, $CFSE^{+}$ and $CFSE^{neg}$ cells were sorted by FACS and co-cultured with murine MAIT "reporter" cells obtained by isolating splenocytes from double transgenic mice (Vα19 and Vβ8 TCR, $C\alpha^{-/-}$, $MR1^{-/-}$). After 16h in absence or presence of 5-OP-RU (100nM), expression of CD69 and CD25 was measured on MAIT reporter cells ($huCD3^{neg}$, $B220^{neg}$, $TCR\beta^{+}$ MR1-tetramer^{+} cells) as shown on the representative example of the gating strategy.

A.

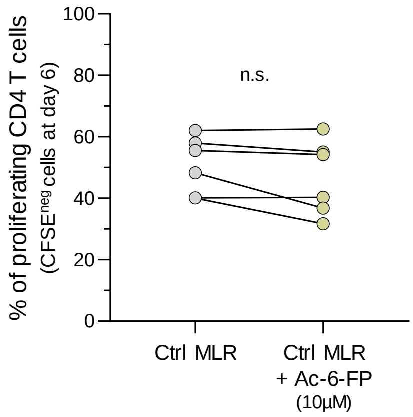

Suppl Fig. 6: Ac-6-FP alone has no impact on alloproliferation in the MLR (related to Fig.2D)

Responder CFSE-labeled CD4 T cells were cultured with stimulator allogeneic CD3-negative PBMCs in absence or presence of 10µM Ac-6-FP and percent of proliferating ($CFSE^{neg}$) cells among CD4 T cells was assessed at day 6 of the MLR.

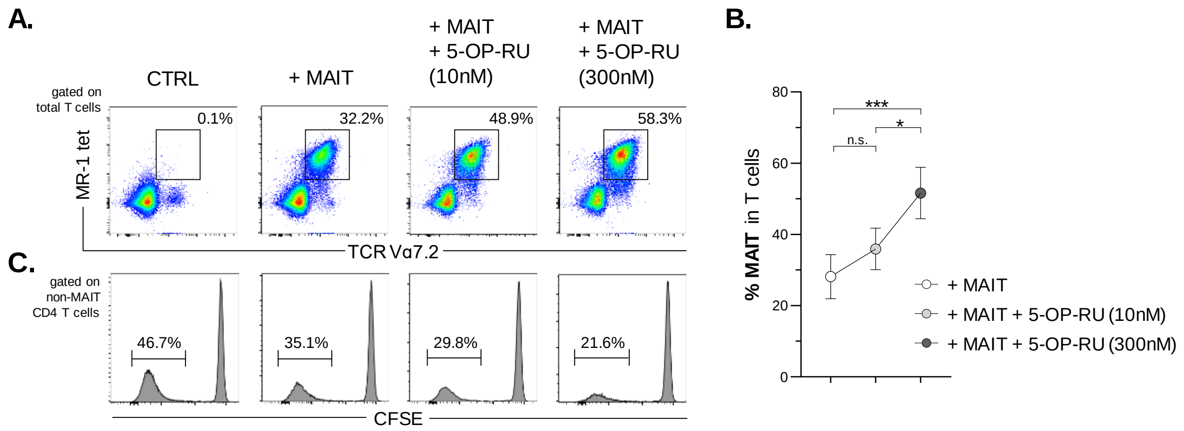

Suppl Fig. 7: MAITs suppressive function is preserved in presence of 5-OP-RU in the MLR.

(related to Fig.2G)

Responder CFSE-labeled CD4 T cells were cultured with stimulator allogeneic CD3-negative PBMCs in absence or presence of 5-OP-RU at indicated concentrations. (**A-B**) MAIT cell proportion among total T cells and (**C**) representative CFSE dilution analysis in (non-MAIT) CD4 T cells (used for data shown in Fig. 2G) are shown at the end (day 6) of the MLR. *p<0.05, ***p<0.001, from one-way ANOVA followed by Tukey's multiple comparisons test.

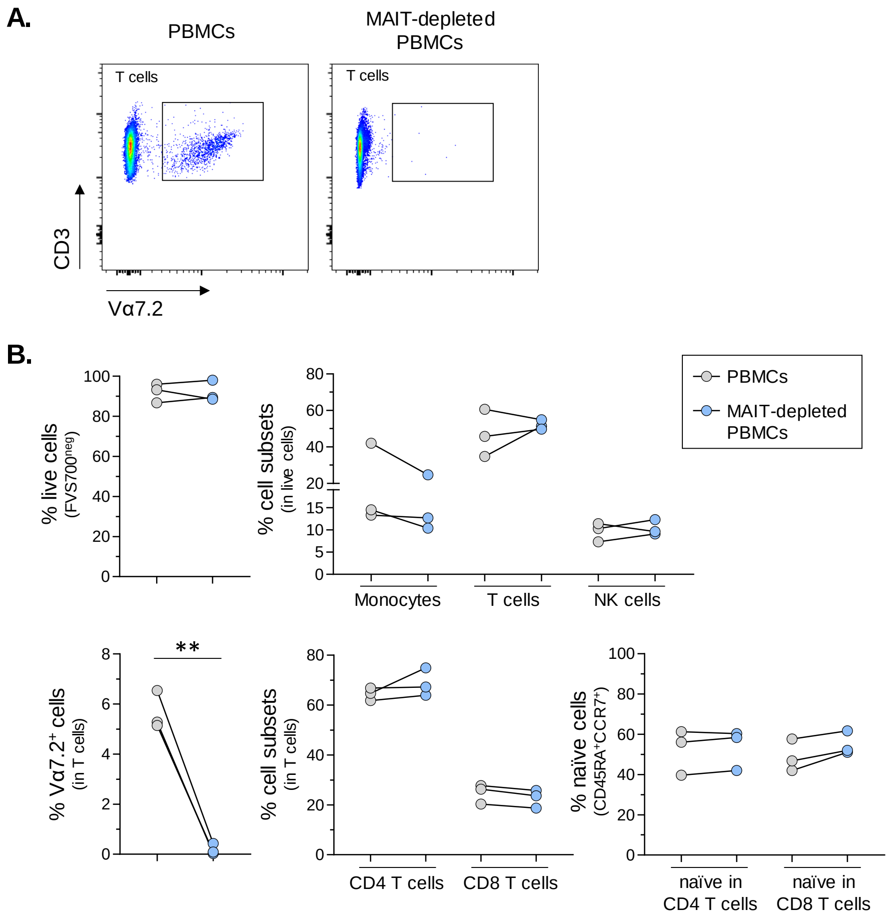

Suppl Fig. 8: Comparison of cell composition of total and MAIT-depleted PBMCs

PBMCs were depleted in TCRVα7.2^{+} cells (containing MAIT cells) by immunomagnetic cell separation. (**A**) Representative Vα7.2 staining (gated in live CD3^{+} T cells) before and after depletion. (**B**) Cellular composition (frequency of indicated immune cell subsets) were compared between total and MAIT-depleted PBMCs. **p<0.01, from paired t-tests (n=3 healthy donors).

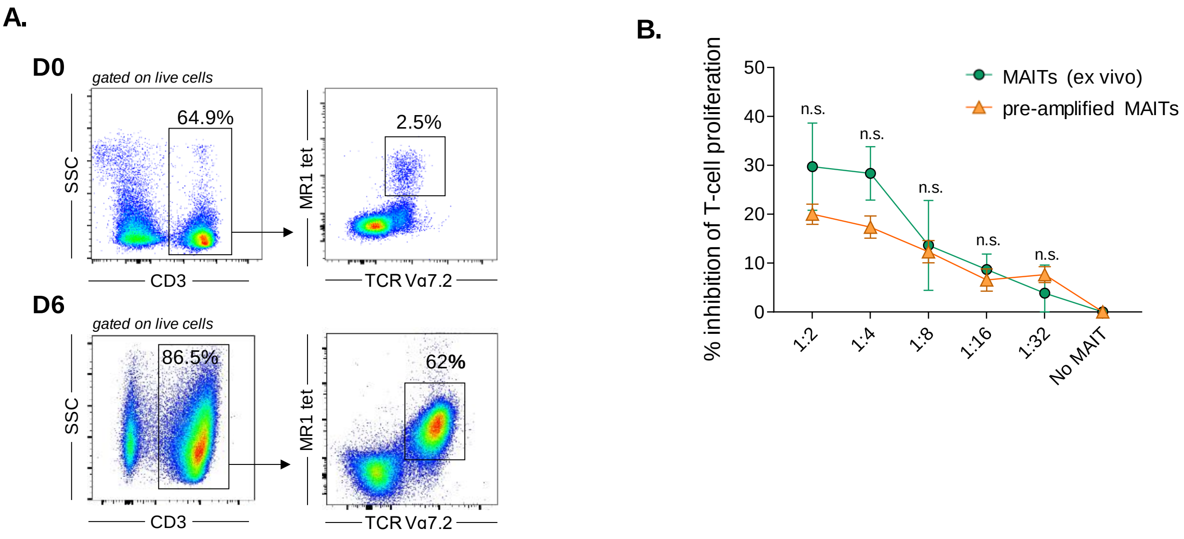

Suppl Fig. 9: Pre-amplified MAIT cells are also able to inhibit proliferation of alloreactive T cells.

MAIT cells were expanded for 6 days from total PBMCs in human T-cell culture medium supplemented with IL-2 (100 U/mL) and 5-OP-RU (300 nM). (**A**) Representative example of MAIT cell expansion from day 0 to day 6 of culture. (**B**) Pre-amplified MAIT cells or freshly ("ex vivo") FACS-sorted purified MAIT cells were added to an MLR with responder MAIT-depleted CFSE-labeled PBMCs co-cultured for 6 days with irradiated allogeneic PBMCs. Summary graph showing the percentage of inhibition of responder T-cell proliferation in the presence of pre-amplified (n=4) or ex vivo purified (n=6) MAIT cells at indicated MAIT: MAIT-depleted PBMC ratios.

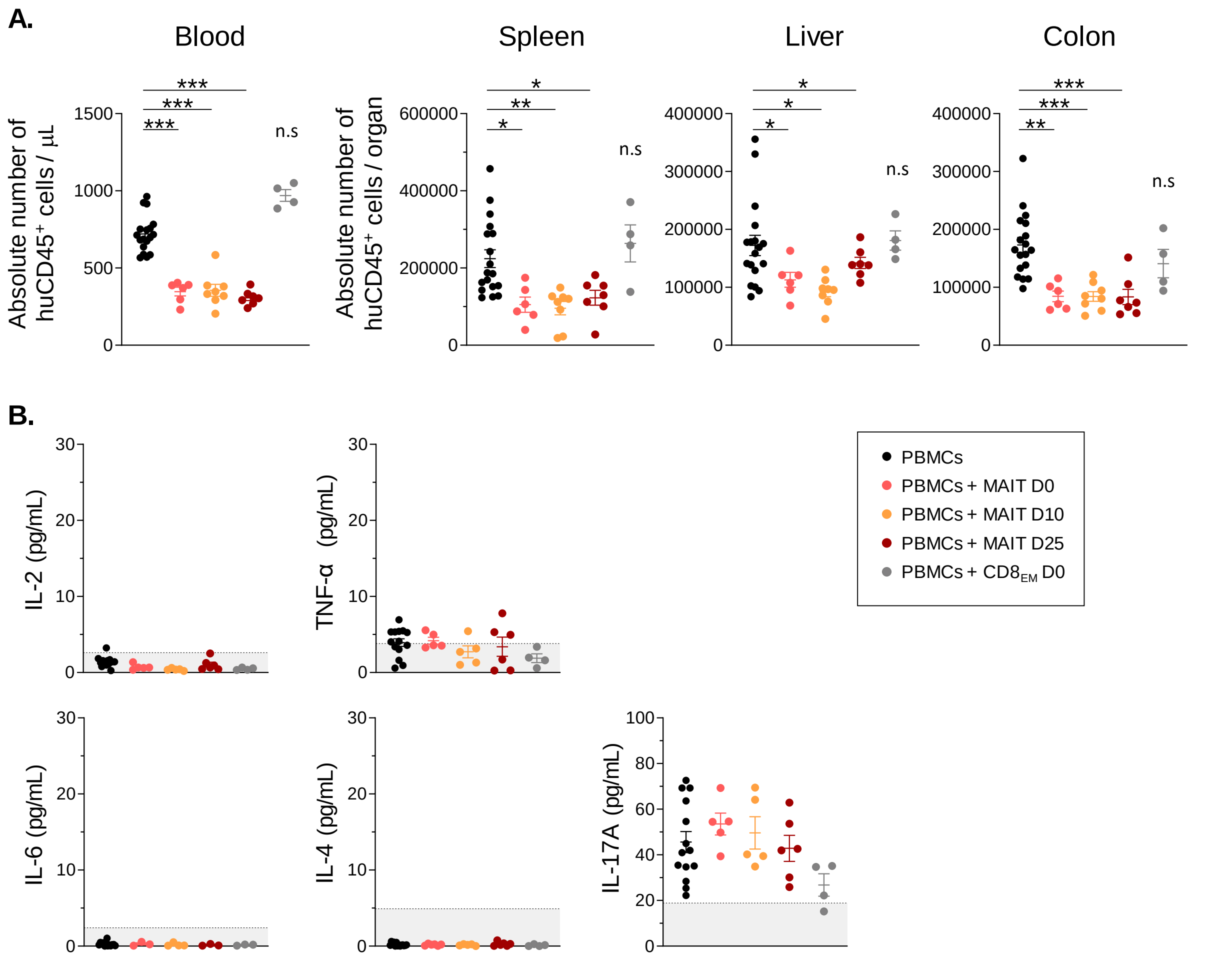

<u>Suppl Fig. 10</u>: MAITs dose-dependently inhibit the proliferation of anti-CD3/CD28-stimulated T cells.

Irradiated (1.3 Gy) NSG mice were injected with 5×10^6 huPBMCs on day 0 without (n=18, black), or with additional transfer of 1×10^6 purified MAITs on day 0 (n=6, red), day 10 (n=8, orange) or day 25 (n=7, brown), or of effector memory CD8 T cells on day 0 (n=4, $CD8_{EM}$, gray). Mice were sacrificed on day 35, and cells from peripheral blood, spleen, liver, and colon were isolated. (**A**) Absolute number of human $CD45^+$ leukocytes ($huCD45^+$) per µL of blood or per indicated organ. (**B**) Circulating levels of IL-2, TNF-α, IL-6, IL-4 and IL-17A quantified by cytometric bead array in mice without (n=14, black), or with transfer of MAITs on day 0 (n=5), day 10 (n=5) or day 25 (n=6), or with transfer of $CD8_{EM}$ cells on day 0 (n=4). Shaded area indicates kit detection threshold for each cytokine. *p<0.05, **p<0.01, ***p<0.001, from one-way ANOVA followed by Tukey's multiple comparisons test.

Suppl Table S1. List of antibodies used in conventional flow-cytometry panels

a. **Panel 1** (surface staining – FACS Celesta)

Marker	Fluorochrome	Manufacturer	Catalog no.	Clone
CFSE	CFSE	ThermoFisher Sci.	C34554	
CD4	BV421	Biolegend	317434	OKT4
CD8	BV650	Biolegend	344730	SK1
CD3	BV785	Biolegend	317330	OKT3
CD161	PE Dazzle594	Biolegend	339940	HP-3G10
TCRva 7.2	BV605	Biolegend	351720	3C10
MR1	APC	Biolegend	361108	26.5
Viability Stain	FVS700	BD Biosciences	564997	

b. **Panel 2** (intracellular staining – FACS Celesta)

Marker	Fluorochrome	Manufacturer	Catalog no.	Clone
CD45	APC Cy7	BD Biosciences	557833	2D1
CD4	BV605	Biolegend	317438	OKT4
CD8	BV650	Biolegend	344730	SK1
CD69	PE	Biolegend	310906	FN50
INF-γ	PE-Cy7	BD Biosciences	560741	4S.B3
perforine	PE-CF594	BD Biosciences	563763	δG9
IL-10	APC	Biolegend	501410	JES3-9D7
IL17	BV421	Biolegend	512322	BL168
TNF-a	BV785	Biolegend	502948	MAb11
Ki67	FITC	Biolegend	151212	11F6
Viability Stain	FVS700	BD Biosciences	564997	

c. **Panel 3** (human cells in mice blood and tissues in the xeno-GVHD model – FACS Celesta)

Marker	Fluorochrome	Manufacturer	Catalog no.	Clone
CD45	APC Cy7	BD Biosciences	557833	2D1
CD3	BV785	Biolegend	317330	OKT3
CD4	BV421	Biolegend	317434	OKT4
CD8	BV650	Biolegend	344730	SK1
TCRva 7.2	BV605	Biolegend	351720	3C10
MR1 Tetramere	PE	NIH		
CD25	BV711	Biolegend	302636	BC96
CD127	BV421	BD Biosciences	568139	HIL-7R-M21
CD45RA	PE Cy7	Biolegend	304126	HI100
CD27	APC	Biolegend	302810	O323
Viability Stain	FVS700	BD Biosciences	564997	

d. **Panel 4** (surface staining – FACS ARIA)

Marker	Fluorochrome	Manufacturer	Catalog no.	Clone
CFSE	CFSE	ThermoFisher Sci.	C34554	
CD8	BV650	Biolegend	344730	SK1
CD4	PECY7	Biolegend	300512	RPA-T4
CD3	BV785	Biolegend	317330	OKT3
CD161	PE Dazzle594	Biolegend	339940	HP-3G10
TCRva 7.2	BV605	Biolegend	351720	3C10
Viability Stain	FVS700	BD Biosciences	564997	
CD223 (LAG-3)	BV711	Biolegend	369320	11C3C65

e. **Panel 5** (intracellular staining – FACS ARIA)

Marker	Fluorochrome	Manufacturer	Catalog no.	Clone
CFSE	CFSE	ThermoFisher Sci.	C34554	
CD8	BV650	Biolegend	344730	SK1
CD4	PECY7	Biolegend	300512	RPA-T4
CD3	BV785	Biolegend	317330	OKT3
CD161	PE Dazzle594	Biolegend	339940	HP-3G10
TCRva 7.2	BV605	Biolegend	351720	3C10
Annexin V	PE	BD Biosciences	556421	
Viability Stain	FVS700	BD Biosciences	564997	
IL-10	BV711	Biolegend	564050	JES3-9D7
CCR7	APC Cy7	Biolegend	353212	G043H7
Nur77	AF647	BD Biosciences	566735	12.14

f. **Panel 6** (MAITs and $CD8_{EM}$ T cells sorting – FACS ARIA)

Marker	Fluorochrome	Manufacturer	Catalog no.	Clone
CCR7	APC	R&D	FAB197A	150503
CD8	BV650	Biolegend	344730	SK1
CD4	BV421	Biolegend	317434	OKT4
CD3	BV785	Biolegend	317330	OKT3
MR1 Tetramere	PE	NIH		
TCRva 7.2	BV605	Biolegend	351720	3C10
Viability Stain	FVS700	BD Biosciences	564997	
CD45RA	PE Cy7	Biolegend	304126	HI100

Suppl Table S2. List of antibodies used in the spectral flow-cytometry panel (AURORA)

Marker	Detector	Fluorochrome	Manufacturer	Catalog no.	Clone
CD3	R8	APC Fire810	Biolegend	344858	SK7
CD4	YG2	CF568	Biotium	BNC680206-500	C4/206
CD8	V7	BV510	Biolegend	300934	HIT8a
CD14	R3	Spark NIR 685	Biolegend	367150	63D3
CD16	UV7	BUV496	BD Biosciences	612944	3G8
CD19	UV9	BUV563	BD Biosciences	612916	SJ25C1
CD56	UV14	BUV737	BD Biosciences	612766	NCAM16.2
CD28	V10	BV605	BD Biosciences	742527	L293
CD25	YG9	PE Cy7	BD Biosciences	557741	M-A251
CD27	B10	PerCP eFluor710	ThermoFisher Sci.	46-0279-42	O323
CD161	YG5	PE Cy5	BD Biosciences	551138	DX12
CD127	R4	APC R700	BD Biosciences	565185	HIL-7R-M21
TCRva 7.2	B9	BB700	BD Biosciences	749483	OF-5A12
CD86	V3	Pacific blue	Biolgend	305423	IT2.2
HLA-DR	V8	BV570	Biolegend	307638	L243
CD39	V4	BV480	BD Biosciences	746454	TU66
MR1	R1	APC	Biolegend	361108	26,5
CD279 (PD-1)	V1	BV421	BD Biosciences	562516	EH12
CD274 (PDL-1)	V11	BV650	BD Biosciences	563740	MIH1
CTLA-4	V15	BV786	BD Biosciences	563931	BNI3
CD223 (LAG-3)	V7	eFluor506	ThermoFisher Sci.	69-2239-42	3DS223H
CD134 (OX40)	YG3	PE Dazzle594	Biolegend	350020	Ber-ACT35 (ACT35)
CD252 (OX40L)	R4	AF700	R&D systems	FAB10541N	159403
CD40	V14	BV750	BD Biosciences	746948	5C3
CD154 (CD40L)	UV16	BUV805	BD Biosciences	748982	TRAP1
CD366 (TIM-3)	V13	BV711	Biolegend	345024	F38-2E2
B7H6	R2	AF647	BD Biosciences	566733	1A5
CD337 (NKp30)	UV11	BUV661	BD Biosciences	750510	p30-15
CD304 (Nrp-1)	UV10	BUV615	BD Biosciences	751266	U21-1283
CD278 (ICOS)	B9	PerCP Cy5.5	Biolegend	313518	C398.4A
CD275 (ICOSL)	UV2	BUV395	BD Biosciences	743011	2D3
MR1 Tetramere	YG1	PE	NIH		
CFSE	B2		ThermoFisher Sci.	C34554	
Viability (nucleic acid) Stain	V5	SYTOXblue	ThermoFisher Sci.	S34857	
